# Supplementary material for: Analysis and source identification of dust event in Khuzestan province using MODIS and SENTINEL satellite data and HYSPLIT model (2018–2022)
Source: PLoS One. 2026 May 7;21(5):e0348129. doi: 10.1371/journal.pone.0348129 (PMC13152170; doi:10.1371/journal.pone.0348129)
Supplement: Attachment — Submitted filename: Comments.docx [file pone.0348129.s005.docx]

**Comments on the manuscript “Analysis and Source Identification of Dust Pollution in Khuzestan Province Using MODIS and SENTINEL Satellite Data and HYSPLIT Model (2018–2022)"**

The present study examines dust pollution dynamics in Khuzestan from 2018 to 2022 using an integrated approach that combines satellite-derived AOD and Aerosol Absorption Index (AAI), ground-based PM₂.₅ and PM₁₀ measurements, HYSPLIT backward trajectory modeling, wind rose analyses, and wetland area assessments via the Modified Normalized Difference Water Index (MNDWI). The relationship between the monthly Shadegan Wetland area and dust-polluted days in Shadegan city was evaluated using key statistical metrics. Analysis of 2022 data revealed strong correlations between satellite-derived AOD and ground-based PM₂.₅ confirming the reliability of satellite observations. PM₂.₅ concentrations ranged from 40 to 700 µg/m³, and PM₁₀ from 75 to 800 µg/m³, peaking in 2022 due to prolonged drought, wetland desiccation, and transboundary dust inflows. Five extreme dust storms in 2022 (AQI = 500, except Storm 5 at 473) were traced using HYSPLIT and MODIS imagery. Storms 1–4 originated from Turkey, Syria, Iraq, and Egypt, descending below 500 m in Khuzestan, while Storm 5 emerged from southern Iran (Hormozgan, Makran coasts) and the Persian Gulf, rising to 2,500 m before descending below 500 m. Wind rose analyses indicated dominant northwest and west winds (> 5 m/s) carrying transboundary dust, while southern Persian Gulf winds intensified local contributions. In 2022, AOD (0.2–2.2) and AAI (-0.1 to 1.3) peaked along desiccated wetland margins. Mitigation strategies require wetland restoration, desertification control, real-time monitoring, regional cooperation, and public awareness.

The manuscript requires further major modification as per the following points:

**Major Comments**

1. The authors state that they used coding within the Google Earth Engine platform, but the explanation does not clearly describe which algorithms were implemented or how data filtering, cloud masking, and quality control were performed. Furthermore, in Section 2.3, while a scaling factor of 0.001 is mentioned for MODIS data processing, there is no discussion on how missing values, cloud contamination, or aerosol quality flags were handled.
2. The correlation analysis between wetland area and polluted days shows strong relationships (R² = 0.71 for PM2.5 and 0.68 for PM10), yet the manuscript does not specify the sample size used for these calculations. Contradict in y axis in AOD values with respect to the PM2.5 and PM10. It seems like both the sample size are different. According to your study, corelation should be conducted using data from 2018 to 2022, but it was only performed for the year 2022. Why?
3. Several correlation metrics are presented (Pearson, Spearman, MI, DC), but there is no discussion explaining why their strengths differ or what unique insights each metric contributes.
4. The manuscript lacks any discussion of uncertainties associated with satellite retrievals, model simulations, or statistical analyses. AOD retrievals have known uncertainties, HYSPLIT trajectories have positional uncertainties that increase with time, and the MNDWI-based wetland area estimates are subject to classification errors. These uncertainties should be quantified and their potential impact on the study conclusions discussed.
5. Section 1.2 covers recent dust storm studies using HYSPLIT, it lacks discussion of previous work specifically on Khuzestan Province or similar arid regions in Iran. Given that this is a regional study with policy implications, more context about previous dust research in this specific area would strengthen the introduction. Additionally, the review focuses heavily on HYSPLIT applications but gives less attention to literature on satellite-based dust monitoring and wetland-dust relationships.
6. Wind rose section (correct the section no.) analyses for multiple stations, but this analysis feels disconnected from the main narrative. The wind patterns should be more explicitly linked to the HYSPLIT trajectory results and discussed in terms of how local wind conditions interact with transboundary dust transport.
7. The conclusions mention mitigation strategies such as "wetland restoration, desertification control, real-time monitoring, regional cooperation, and public awareness" but provide no specific, actionable recommendations. Given the detailed analysis presented, the manuscript should offer more concrete suggestions: What wetland restoration techniques are most appropriate? What threshold levels should trigger public health warnings? What specific forms of regional cooperation are needed?

**Minor Comments**

1. The abstract is excessively long and contains too much detailed numerical information. According to PLOS ONE guidelines, abstracts should be concise (less than 300 words). The abstract should be restructured to emphasize key findings rather than listing numerous specific values.
2. Figure 3 attempts to show both land cover and station locations but the color scheme makes it difficult to distinguish between different land cover types.
3. The manuscript uses inconsistent terminology for key concepts. For example, "dust storm," "dust event," and "dust pollution" are used interchangeably without clear definitions. Similarly, "wetland desiccation," "wetland shrinkage," and "wetland area reduction" refer to the same phenomenon. Standardizing terminology throughout would improve clarity.
4. The Results and Discussion section presents findings but rarely compares them with previous studies. For instance, the AOD values, dust storm frequencies, and source attribution results should be compared with earlier research on Khuzestan or similar Middle Eastern regions. How do the identified dust sources align with or differ from previous source apportionment studies?
5. Table 1 presents AQI relationships but the "Hazardous" category for AAI (>5) has no corresponding AOD range, marked with a dash. This seems inconsistent. Additionally, the relationship between AAI and AQI appears oversimplified- is this a direct linear relationship or based on empirical studies?
6. The manuscript analyses "daily MODIS imagery" and mentions "360 images per year," but MODIS Terra and Aqua combined can provide twice-daily coverage. Clarify whether you used Terra only, Aqua only, or combined products, and explain how multiple daily observations were aggregated.
7. Dust Storm No. 5 reveals an interesting pathway through the Persian Gulf and southern Iran, yet this finding is not adequately explored. The manuscript mentions "evaporation processes and surface moisture" but does not quantify these effects or discuss the mechanism by which the Gulf modifies dust transport. This deserves more detailed investigation and cite the references that validate this point.
8. The selection of a 48-hour backward trajectory requires justification, as longer trajectories could help identify more distant source regions.
9. Correct the grammatically error in the whole manuscript especially in line no. 683-699.
