# Supplementary material for: Analysis and source identification of dust event in Khuzestan province using MODIS and SENTINEL satellite data and HYSPLIT model (2018–2022)
Source: PLoS One. 2026 May 7;21(5):e0348129. doi: 10.1371/journal.pone.0348129 (PMC13152170; doi:10.1371/journal.pone.0348129)
Supplement: Attachment — Submitted filename: Response to reviewers.docx [file pone.0348129.s006.docx]

**Authors' Response to the Reviewers' Comments**

**Submission ID:** PONE-D-25-51186

**Title**: Analysis and Source Identification of Dust Pollution in Khuzestan Province Using MODIS and SENTINEL Satellite Data and HYSPLIT Model (2018–2022)

Dear Editor,

Thank you for providing us the opportunity to revise and resubmit our manuscript, *“Analysis and Source Identification of Dust event in Khuzestan Province Using MODIS and SENTINEL Satellite Data and HYSPLIT Model (2018–2022)”*, for publication in your esteemed journal. We sincerely appreciate the time and effort you and the reviewers have invested in evaluating our work. Their detailed feedback and constructive suggestions have greatly enhanced the quality of our manuscript.

We have carefully reviewed and addressed all the reviewers’ comments and suggestions. Our responses are organized systematically, detailing the changes made to the manuscript to incorporate each suggestion. Below, we provide a comprehensive summary of our revisions and responses:

Editor#

**Comment 1.** Please ensure that your manuscript meets PLOS ONE’s formatting and style requirements, including correct file naming. Refer to the PLOS ONE style templates: Main Body Template, Title/Authors/Affiliations Template.

**Response:** Thanks. The manuscript and file naming have been revised to fully comply with the PLOS ONE formatting and style guidelines.

**Comment 2.** PLOS ONE has specific guidelines on code sharing for studies in which author-generated code supports the findings. You must ensure your code is made openly available without restrictions and follows best practices for reproducibility and reuse. Guidelines: Code Sharing Policy.

**Response:** Thank you for your valuable comment regarding code sharing. We have prepared the Google Earth Engine scripts as text files and uploaded them as Supplementary Files along with the revised manuscript. This ensures that the code is available without restrictions, facilitating reproducibility and reuse in accordance with PLOS ONE guidelines.

**Comment 3.** Please clarify the role of funders in your study. If funders had no role, include this exact statement:

“The funders had no role in study design, data collection and analysis, decision to publish, or preparation of the manuscript.” Include this corrected “Role of Funder” statement in your cover letter.

**Response:** Thank you, the issue has been corrected, and the role of the funders has been clearly stated in the revised manuscript and cover letter. The following statement has been included as requested.

**Comment 4.** Confirm whether your submission includes all raw data necessary to replicate your results (“minimal data set”). If not, upload missing data as Supporting Information or deposit them in a public repository (provide DOI or URL). If there are restrictions, explain who imposed them and how others can request access

**Response:** Thanks. The corresponding codes used in this study will be provided, through which all satellite images and remote sensing data can be downloaded. Some datasets were obtained from the HYSPLIT model, which is publicly available at the following link: <https://www.ready.noaa.gov/HYSPLIT.php>. MODIS True Color imagery was downloaded from NASA’s public platform at <https://worldview.earthdata.nasa.gov>. Ground-based air quality (PM₂.₅ and PM₁₀) data were obtained from the Iranian National Air Quality Monitoring System, which provides daily accessible data via <https://aqms.doe.ir/Home/AQI>. Wind speed and direction data were acquired from the Iran Meteorological Organization (<https://data.irimo.ir/>) through an official data request submitted by the university. For data access coordination, inquiries can be made by contacting the Meteorological Organization at +98-21-66070017-19 (extension 122, Ms. Asadi).

**Comment 5.** Figures 3, 5, 6, 7, 8, 9, 10, 11, 12, 16, and 17 may contain copyrighted map images. PLOS ONE only accepts figures under CC BY 4.0 license. You must either: a) Obtain written permission using the PLOS content permission form, or b) Replace/remove copyrighted figures and ensure replacements comply with CC BY 4.0. If replaced, specify in captions whether new figures are “similar but not identical” and provide the data source (e.g., NASA, USGS, Natural Earth)

**Response:** Thanks. All the mentioned figures have been revised, and the related statements regarding data sharing and copyright compliance have been added to each figure and caption. All figures now fully adhere to the CC BY 4.0 license requirements.

**Comment 6.** Please include Table 3, which is referenced on page 34 of your manuscript, but is missing in the submitted files.

**Response:** Thanks. Table 3 has been changed to Table 4 and is now placed on page 35 of the revised manuscript.

**Comment 7.** If reviewer comments suggest additional citations, review and cite only relevant works. Citations are not mandatory unless the editor explicitly requires them.

**Response:** Thanks. Several references have been added to the manuscript based on the reviewer’s suggestions to enhance the literature review, materials and methods section, and discussion. Approximately 27 references have been included, as highlighted in yellow for Reviewer 1 (to improve the introduction and literature review), blue for Reviewer 2 (to provide references for tables), purple for Reviewer 3 (to enrich the literature review and discussion), and green for the methods section (which previously lacked citations). These references are as follows:

Middleton, N. J. (2017). Desert dust hazards: A global review. *Aeolian Research*, *24*, 53–63. <https://doi.org/10.1016/j.aeolia.2016.12.001>

Kok, J. F., Storelvmo, T., Karydis, V. A., Adebiyi, A. A., Mahowald, N. M., Evan, A. T., He, C., & Leung, D. M. (2023). Mineral dust aerosol impacts on global climate and climate change. *Nature Reviews Earth & Environment*, *4*(2), 71–86. <https://doi.org/10.1038/s43017-022-00379-5>

Khanfari, V., Asgari, H. M., & Dadollahi-Sohrab, A. (2024). Forecasting wetland transformation to dust source by employing CA-Markov model and remote sensing: A case study of Shadgan International Wetland. *Wetlands*, *44*(6), Article 96. <https://doi.org/10.1007/s13157-024-01856-x>

Eskandari Damaneh, H., Khosravi, H., Habashi, K., Eskandari Damaneh, H., & Tiefenbacher, J. P. (2022). The impact of land use and land cover changes on soil erosion in western Iran. *Natural Hazards*, *110*(3), 2185–2205. <https://doi.org/10.1007/s11069-021-05032-w>

yousefi kebriya, A., Khalili, A. and Rezaei, H. (2025). Monitoring and analysis of dust storm trajectories in Sistan and Baluchestan Province using MODIS satellite products and the HYSPLIT model. *Iranian Journal of Remote Sensing and GIS*, (), -. doi: 10.48308/gisj.2025.238735.1252

Shirgholami, M., Rousta, I., Olafsson, H., Petracchini, F., & Krzyszczak, J. (2025). Tracing the dust: Two decades of dust storm dynamics in Yazd Province from ground-based and satellite aerosol observations. Atmosphere, 16(11), Article 1242. <https://doi.org/10.3390/atmos16111242>

Soleimani Sardo, F. and Krakauer, N. (2025). Dust Modeling using MODIS Data and WRF-Chem and HYSPLIT Models (Case Study of Dust Storm from December 16 to 20, 2016). *Sustainable Earth Trends*, (), -. doi: 10.48308/set.2025.238019.1098

Yousefi-Kebriya, A., Nadi, M., Afaridegan, E., & Sun, Z. (2025). Wetland shrinking and dust pollution in Khuzestan Iran: Insights from sentinel-5 and MODIS satellites. *Scientific Reports*, *15*(1), Article 13626. <https://doi.org/10.1038/s41598-025-96935-2>

Rangzan, K. and balouei, F. (2024). Dust monitoring and investigation of its relationship with topographical, climatic and vegetation factors. *Desert Ecosystem Engineering*, *12*(39), 43-60. doi: ‎10.22052/deej.2024.253713.1025

Vatanparast Ghaleh Juq, F., Salahi, B. and Zeinali, B. (2024). Monitoring Temporal-Spatial Changes of Atmospheric Suspended Dust in Selected Provinces of the Western Half of Iran Using MODIS and Sentinel-5 Images. *Geography and Environmental Planning*, *35*(3), 113-128. doi: 10.22108/gep.2024.140621.1635

Noroozi, A. and Shoaei, Z. (2018). Identifying areas with dust generation potential in the south west of Iran, case study: Khuzestan Province. *Watershed Engineering and Management*, *10*(3), 398-409. doi: 10.22092/ijwmse.2018.117329

Arami, S. H. , Karimi Sangchini, E. , Alimahmoodi Sarab, S. , Dinarvand, M. and Yasrebi, B. (2024). Mapping spatial and temporal pattern of risk and hazard of dust storms in Khuzestan province. *Journal of Arid Biome*, *14*(1), 61-78. doi: 10.29252/aridbiom.2024.21433.2007

Farzanehpey,F. , Ranjbar-Fordoe,A. , Khosravi,H. and Mosavi,S. H. (2024). Evaluation of dust changes and its relationship with temperature (Case study: Khuzestan province). *Integrated Watershed Management*, *4*(1), 16-29. doi: 10.22034/iwm.2024.2014553.1112

Shayeghannoor,F. and Ahmadi,M. (2025). Statistical analysis of dust hazard changes in southwest Iran (Khuzestan province). *Journal of Climate Research*, *1403*(59), 91-107. doi: 10.22034/jcr.2025.202092

Dargahian, F., Gohardoust, A., Lotfinasabasl, S. and Teimouri, S. (2024). Determining the contribution of neighboring countries in the emission of dust to Khuzestan province. *Iranian Journal of Range and Desert Research*, *31*(1), 74-92. doi: 10.22092/ijrdr.2024.131464

Zarasvandi, A., Carranza, E. J. M., Moore, F., & Rastmanesh, F. (2011). Spatio-temporal occurrences and mineralogical–geochemical characteristics of airborne dusts in Khuzestan Province (southwestern Iran). *Journal of Geochemical Exploration*, *111*(3), 138–151. <https://doi.org/10.1016/j.gexplo.2011.04.004>

MalAmiri, N., Rashki, A., Al-Dousari, A., & Kaskaoutis, D. G. (2025). Socioeconomic and health impacts of dust storms in southwest Iran. *Atmosphere*, *16*(2), Article 159. <https://doi.org/10.3390/atmos16020159>

Mohammadi, Z., Rahimi, D., Najafi, M. R., & Zakerinejad, R. (2024). The impact of environmental degradation and climate change on dust in Khuzestan province, Iran. *Natural Hazards*, *120*(6), 4329–4348. <https://doi.org/10.1007/s11069-023-06368-1>

Gretton, A., Bousquet, O., Smola, A., & Schölkopf, B. (2005). Measuring statistical dependence with Hilbert–Schmidt norms. In S. Jain, H. U. Simon, & E. Tomita (Eds.), Algorithmic learning theory (pp. 63–77). Springer. https://doi.org/10.1007/11564089_7

Mansournia, M. A., Waters, R., Nazemipour, M., Bland, M., & Altman, D. G. (2021). Bland–Altman methods for comparing methods of measurement and response to criticisms. Global Epidemiology, 3, 100045. https://doi.org/10.1016/j.gloepi.2020.100045

Yousefi Kebriya,A. , Shiukhy Soqanloo,S. and Yousefi Kebriya,H. (2025). Monitoring and Analysis of the Relationship Between Vegetation Cover and Air Pollution Using Sentinel and MODIS Satellite Data in Mazandaran Province (2019–2024). (e231767). *Iranica Journal of Energy & Environment*, (), e231767

Zarei, S., Karbassi, A., Sadrinasab, M., & Sarang, A. (2023). Investigating heavy metal pollution in Anzali coastal wetland sediments: A statistical approach to source identification. Marine Pollution Bulletin, 194, 115376. https://doi.org/10.1016/j.marpolbul.2023.115376

Razeghi, N., Hamidian, A. H., Abbasi, S., & Mirzajani, A. (2024). Distribution, flux, and risk assessment of microplastics at the Anzali Wetland, Iran, and its tributaries. Environmental Science and Pollution Research, 31, 54815–54831. https://doi.org/10.1007/s11356-024-27874-7

Yarramsetty, S., Siva Kumar, M., & Anand Raj, P. (2023). Enhancing comfort in tropical institutional buildings: Integrating thermal, acoustic and visual performance with a unified index. International Journal of Engineering, 36(12), 2253–2263. https://doi.org/10.5829/IJE.2023.36.12C.15

Ghomeshion, M., Vali, A. A., Ranjbar Fordoei, A., & Mousavi, S. H. (2022). Investigating the effect of land cover on dust spatial distribution in Southern Khuzestan province. ECOPERSIA, 10, 179–189. https://doi.org/10.1001.1.23222700.2022.10.3.2.9

Gong, H., Li, Y., Zhang, J., Zhang, B., & Wang, X. (2024). A new filter feature selection algorithm for classification tasks by ensembling the Pearson correlation coefficient and mutual information. Engineering Applications of Artificial Intelligence, 131, 107865. https://doi.org/10.1016/j.engappai.2024.107865

Xie, H., Zhang, L., Lim, C. P., Yu, Y., Liu, C., Liu, H., & Walters, J. (2019). Improving K-means clustering with enhanced Firefly Algorithms. Applied Soft Computing, 84, 105763. https://doi.org/10.1016/j.asoc.2019.105763

**Comment 8.** While your manuscript addresses an important and relevant topic, the reviewers have raised serious concerns that must be resolved before further consideration.

Reviewer #2 observed that several figures and tables in your manuscript appear nearly identical to those in Yousefi-Kebriya et al., 2025 (Scientific Reports).

This raises a potential issue of data duplication and lack of originality, which would be unacceptable for publication in PLOS ONE if confirmed. You must provide a clear and detailed explanation regarding this issue, including: The provenance (origin) of all datasets, figures, and tables; Whether any materials were reused from previously published work; How the current data, period, or methodology differ from those in prior publications.

**Response:** We sincerely thank the editor for raising this important concern. We would like to clarify that all analyses, datasets, figures, and tables in the current manuscript are original and distinct from previously published work, including Yousefi-Kebriya et al., 2025 (Scientific Reports). While the previous studies focused on specific aspects of dust pollution, the present research introduces several new elements that have not been addressed before. These include:

1. A comprehensive five-year (2018–2022) analysis of dust events in Khuzestan Province integrating multiple satellite datasets (MODIS, Sentinel-2, Sentinel-5), ground-based PM₂.₅ and PM₁₀ measurements, and HYSPLIT trajectory modeling.
2. Introduction of new aerosol indices (AOD Max and AOD Sum) to assess peak intensity and cumulative dust load over time.
3. A detailed investigation of both local and transboundary dust sources, including a 24-hour lag analysis between dust from Iraq and air quality in Ahvaz, using advanced statistical metrics such as PCA, K-Means clustering, Bland–Altman analysis, Mutual Information, and Distance Correlation.
4. In this study, the Modified Normalized Difference Water Index (MNDWI) was employed to quantify wetland area changes, providing a novel approach compared to previous studies.”

We assure that none of the figures or tables in the manuscript are similar to other published works or studies. Regarding Figure 1, which Reviewer #2 mentioned, it has been modified due to changes in the materials and methods and the addition of several new analyses, and it is not similar to any other research. Figure 2 was generated using custom coding in Google Earth Engine, incorporating Landsat and MODIS satellites and a Random Forest approach, and no similar version exists in other publications. Based on the reviewer’s comments regarding color, structure, and station identification, this figure has also been updated. Table 1, which was highlighted by Reviewer #2, was compiled from various articles and official site instructions (with proper citations) and has been further revised to address these concerns.

In summary, this manuscript presents a significantly expanded and original study, integrating multiple novel analyses and data sources.

**Reviewer #1**

**Comment 1.** L52 ‘global challenge with far-reaching consequences for ecosystems, human health, and climate systems (Siddiqua et al. 2022).’ Two comments: a) the Siddiqua et al. 2022 paper is not about dust storms! b) there are many more consequences for human society that should be mentioned, sectors impacted include agriculture, transport, power generation (see eg https://doi.org/10.1016/j.aeolia.2016.12.001), for climatic impacts see eg <https://doi.org/10.1038/s43017-022-00379-5>

**Response:** We sincerely thank the reviewer for this insightful comment. Following the reviewer’s recommendation, the incorrect citation to Siddiqua et al. (2022) has been removed and replaced with more relevant references. In addition, a broader description of the societal and climatic impacts of dust storms has been incorporated. The modifications have been highlighted in yellow from lines 37 to 39 of the revised manuscript.

Dust events, a severe form of atmospheric pollution originating in arid and semi-arid regions, have emerged as a global environmental challenge with wide-ranging consequences for ecosystems, human health, agriculture, transportation, and power generation [1], as well as significant radiative and climatic impacts [2].

**Comment 2.** L53-55 ‘increasing frequency and intensity of these storms’ IN SOME PARTS OF THE WORLD. Not all. In others frequency and intensity have declined in recent times.

**Response:** Thanks, the sentence has been revised to clarify that the increasing frequency and intensity of dust storms occur only in certain regions of the world, while other areas have experienced a decline in recent years. This modification addresses the regional variability of dust activity. The changes are highlighted in yellow from lines 39 to 42 of the revised manuscript.

In several regions of the world, the increasing frequency and intensity of dust events have been linked to climate change and human activities, while in other areas, their occurrence has shown a declining trend in recent years, reflecting strong regional variability in dust dynamics.

**Comment 3.** L 59-67 I would delete this paragraph. It is very generalised, selective and adds little to the paper. In its place, I want to see a review of some of the numerous other papers on dust storms affecting Khuzestan (including papers documenting the ‘traditional perspective that attributes most of Khuzestan’s dust storms to Iraqi and Syrian deserts’ as cited at L661, and papers that identify the Shadegan wetland as a dust source, eg https://doi.org/10.1007/s13157-024-01856-x).

**Response:** Thanks. Following the recommendation, the original paragraph (L59–67) has been fully deleted and replaced with a more focused discussion on dust storms affecting Khuzestan Province, highlighting the role of the Shadegan Wetland as an internal dust source. Relevant literature and quantitative data on land-cover change and soil erosion have been incorporated. The revisions have been highlighted in yellow from lines 45 to 54 in the revised manuscript.

Wetlands are disappearing globally at alarming rates, with 71% of wetlands converted to other land-cover types since 1900 [5]. In southwestern Iran, the Shadegan Wetland has been increasingly exposed to drought and land-cover changes, causing previously inundated areas to dry out and become active dust sources. Remote sensing and supervised classification analyses of Landsat TM, ETM, and OLI images for 1989, 2003, and 2017 indicate that while agricultural land, wetlands, water bodies, and built-up areas increased between 1989 and 2003, bare lands and wetland vegetation decreased, enhancing soil erosion. Between 2003 and 2017, bare lands, water bodies, and built-up areas further increased, whereas agricultural lands, wetlands, and wetland vegetation decreased, with soil erosion rates rising from 45.56% in 1989 to 52.24% in 2017. These results indicate that land-cover changes, particularly wetland degradation, have made the Shadegan Wetland a major internal source of dust in Khuzestan Province [6].

**Comment 4.** 1.2. Review of previous studies: this is a curious assortment of studies. I suggest the authors focus only on those relevant to this paper (i.e studies using MODIS and SENTINEL data and the HYSPLIT Model). Hence delete Alebic-Juretic et al. (2023), Schiavo et al. (2023) and others with little relevance to this paper.

**Response:** Thanks. Following the recommendation, all irrelevant references, including Alebic-Juretic et al. (2023) and Schiavo et al. (2023), have been removed. Several studies directly relevant to the use of MODIS, SENTINEL, and HYSPLIT in dust storm analysis have been added to strengthen the review. These revisions have been highlighted in yellow in lines 86–92, 100–103, and 103–108 of the revised manuscript.

Yousefi Kebriya et al. [16] analyzed dust indices in Sistan and Baluchestan (2018–2023) using MODIS data in Google Earth Engine and HYSPLIT backward trajectories. TDI rose from 0.0055 (2018–2019) to 0.0058 (2021), then fell to 0.0056 (2022) and 0.0057 (2023; min. threshold 0.018), identifying Zabol, Iranshahr, Khash, and Chabahar as key internal sources. AOD-Max peaked at 3.5 (2022, hazardous) and 3.1 (2023, very hazardous) in eastern/southern borders; AOD-Sum indicated >100 dusty days/year in east/northeast and >120 in south, averaging 1.9 (2023, hazardous). HYSPLIT revealed dust origins in Turkmenistan/Afghanistan deserts, reaching Zabol in 24 h (>1500 m to <500 m altitude) and Zahedan in 48 h.

Shirgholami et al. [18] examined dust event trends in Yazd province, Iran (2003–2022), using synoptic station data (~0.5 dusty days/year increase, peaking March–May) and MODIS AOD (rising annual load, May maximum). Central/northern regions were most affected, driven by drought, desertification, wetland drying, land use changes, and transboundary dust from Iraq/Syria/Saudi Arabia.

Soleimani Sardo et al. [19] investigated a December 16–20, 2016 dust event in Iran using MODIS AOD, WRF-Chem, and HYSPLIT models. HYSPLIT forward trajectories showed particles from Jazmurian wetland moving southward, with AOD peaking at 0.8 in the region. Significant AOD increases occurred in southern Sistan-Baluchestan, Afghanistan-Pakistan border, west Oman Sea, and east Persian Gulf, highlighting Jazmurian’s emerging role as a major fine-dust source due to reduced wetland inflow from climatic and anthropogenic factors.

**Comment 5.** 2.1. Study Area should come before 1.3. Objectives and innovations of the study.

**Response:** Thanks. In Section 1.1, the information was added at the end to help readers better understand Khuzestan province and its location, while in Section 2.1, the study area is described separately. The changes are highlighted in yellow from lines 71 to 79.

Khuzestan province, located in southwestern Iran along the border with Iraq, encompasses extensive desert areas and a diverse range of land uses, including agricultural lands, urban areas, and natural landscapes. The province also hosts two major wetlands, Shadegan and Hoor al-Azim, which are crucial for local hydrology, biodiversity, and ecosystem services. Over recent decades, these wetlands have experienced significant shrinkage due to prolonged drought and changes in water management, exposing large areas of dry land that act as major dust sources. Despite the ecological and economic importance of Khuzestan, detailed studies on the dynamics of dust events originating from these local sources remain limited. Consequently, the province has become one of the most affected regions in Iran by dust event, with notable impacts on air quality and public health (Hosseini et al., 2020).

**Comment 6.** L180-187 delete from ‘This province is situated in the southwest of Iran………….’ to ‘…along the shores of the Persian Gulf.’ All this information is on the map or irrelevant.

**Response:** Thanks. The suggested edits have been made, and the redundant information has been removed. The changes are highlighted in yellow in lines 195–196.

Khuzestan Province is located between 29.95° to 33.55° N and 47.66° to 50.55° E, covering 9.3% of Iran, as shown in Figure 2.

**Comment 7.** L196 how many monitoring stations?

**Response:** Thanks, according to the Air Quality Monitoring System of Iran (https://aqms.doe.ir/Home/AQI), Khuzestan Province has 31 monitoring stations. However, for the 5-year period considered in this study (2018–2022), data from 22 stations were used, with their locations shown in Figure 2. The text has been revised accordingly, and the changes are highlighted in yellow in lines 205–211.

This research utilized data on particulate matter (PM2.5 and PM10) for a 6-year period (2018–2023) from 22 air pollution monitoring stations in Khuzestan Province. To assess the impact of dust events originating from Iraq on Khuzestan Province—particularly on the city of Ahvaz—19 virtual stations were designed and positioned outside Iran’s western borders, in Iraq and its surrounding areas, to examine the 24-hour lag effect and its influence on dust transport toward Khuzestan. Among the real stations, data from Shadegan, Ahvaz, Abadan, Bostan, Gotvand, and Mahshahr were used to generate wind roses and analyze wind direction and speed patterns to better understand pollution dispersion, as shown in Figure 2.

**Comment 8.** L224 where does the Air Quality Index (AQI) come from? (Yousefi Kebriya, 2023) is not in the references.

**Response:** Thanks, the Air Quality Index (AQI) is a standardized indicator used to quantify the level of air pollution and its potential effects on human health. In Iran, the AQI is applied to assess air quality across different provinces, including Khuzestan, based on measurements of key pollutants such as PM2.5, PM10, NO₂, SO₂, CO, and O₃. The relevant reference has now been added, and the changes are highlighted in yellow at line 257.

**Comment 9.** L 254 Wind does not form dust. Wind is a critical meteorological factor influencing the EMISSION and dispersion of dust.

**Response:** Thanks, the sentence has been revised according to your suggestion, and the changes are highlighted in yellow in lines 285–286.

Wind is a critical meteorological factor influencing the emission and dispersion of dust. It plays a significant role in transporting dust from dry, uncovered areas to other regions.

**Comment 10.** L 364 ‘variations clearly indicate an increase in dust pollution levels, particularly in PM10, during 2022’ do they? Increase relative to when? The logic is strange.

**Response:** Thanks, we acknowledge that the original sentence referred solely to the year 2022, and following your suggestion, it has been removed to improve the clarity and precision of the manuscript.

**Comment 11.** L754 Figure 13. Does not show a trend, just variation in wetland area.

**Response:** Thanks, the changes have been made, and the figure title has been revised to “Variations in the Shadegan Wetland area (Km²) during the period 2018–2023”, which is highlighted in yellow in line 760.

**Figure 12.** Variations in the Shadegan Wetland area (Km²) during the period 2018–2023

**Comment 12.** L840-863 and Figure 15: these stations need to be labelled on the study area map – most readers will not know where they are.

**Response:** Thanks, the study area and all monitoring stations have now been labeled in Figure 2 to improve clarity for readers.

**Comment 13.** L 985 ‘the contribution of both internal and external dust sources’ does this mean source inside Iran and from other countries?

**Response:** Thanks. Yes, exactly. By “the contribution of both internal and external dust sources,” we meant dust generated within Iran as well as transboundary dust transported from neighboring countries. This clarification has been explicitly mentioned and highlighted in yellow in Lines 968–973 of the revised manuscript.

The research highlights the significant role of the drying Shadegan Wetland in increasing dust levels, particularly PM2.5, and emphasizes internal dust sources present within Khuzestan. It also identifies dust-prone areas in the province, including the western desert regions and the dry shores of the Shadegan Wetland. Additionally, this study introduces the analysis of AOD-Max and AOD-Sum indices maps for Khuzestan province, filling a gap not addressed in previous research.

**Comment 14.** L1004 ‘the study examined the statistical relationship between the monthly area of the Shadegan Wetland and the number of dust-polluted days in Shadegan city’ but Shadegan city is only mentioned here and in the abstract! It must also appear in the main text!

**Response:** Thanks, the text has been revised, and Shadegan city is now included in the main text within the Study Area section. It is noted that the Shadegan Wetland is adjacent to Shadegan city, and the monitoring station is also nearby, as shown in Figure 2 for the study area. The changes are highlighted in yellow in lines 198 to 199.

Shadegan city, situated in the southern part of the province, lies adjacent to the Shadegan Wetland, which strongly influences local air quality and dust conditions.

**Reviewer #2**

**Comment 1.** This work represents a study aimed at analyzing the relationships between climatic factors, land degradation, and environmental consequences in a specific region. The topic is relevant, being related to climate risks, soil degradation, and ecosystem resilience. Nevertheless, in its current form, the manuscript does not meet the standards of an original research study, which require quantitative rigor, analytical novelty, and data transparency.

**Response:** Thank you very much for your valuable points and constructive criticism, which increased the scientific value of this article. Substantial revisions have been made throughout the manuscript to enhance its scientific rigor and analytical depth. The revised version now includes additional quantitative analyses, clearer data presentation, and improved methodological transparency. All of the reviewer’s constructive suggestions have been carefully considered and incorporated.

**Comment 2.** The novelty is not explicitly demonstrated. The authors largely reiterate well-known relationships (temperature increase → ecosystem degradation) without presenting a new mechanism, approach, or original model. It is also unclear which specific scientific gaps in the existing literature this study addresses.

**Response:** Thanks, the section on research innovation has been revised to clearly highlight the specific novelty and the research gap addressed by this study. The revised text is presented in Section 1.3 (Research Novelty), highlighted in blue between lines 169–190 of the updated manuscript.

This study presents an integrated and multidisciplinary framework to analyze dust event and their environmental drivers in Khuzestan Province. The research introduces a novel combination of multi-satellite data, advanced statistical modeling, and atmospheric trajectory analysis to explore both local and transboundary sources of dust events. Unlike previous studies that primarily relied on single satellite datasets or descriptive analyses, the present work simultaneously utilizes MODIS, Sentinel-5, and Sentinel-2 satellite products. The AOD and AAI were employed to assess the intensity and spatial distribution of aerosols, while the MNDWI (Modified Normalized Difference Water Index) was used to monitor wetland shrinkage and its relationship with dust events. To enhance the temporal and spatial characterization of aerosols, the study introduces new indices—AOD Max and AOD Sum capture both the peak intensity and cumulative load of dust over time. These indices, when analyzed alongside HYSPLIT back-trajectory simulations and MODIS True Color imagery, provided a detailed understanding of dust transport pathways and the contribution of transboundary sources from Iraq, Syria, and Saudi Arabia. Furthermore, a 24-hour lag correlation analysis between AOD values of neighboring countries and Ahvaz was conducted to quantitatively examine the influence of cross-border dust transport—an aspect not previously addressed in regional studies. The research also incorporates a wide range of quantitative and correlation-based analyses, including R², Linear Regression, Kendall’s Tau, Pearson and Spearman correlations, PCA, Distance Correlation (DC), Mutual Information (MI), K-Means Clustering, Hilbert-Schmidt Independence Criterion (HSIC) and Bland–Altman analysis. This comprehensive statistical framework fills a key gap in previous works, which often lacked rigorous quantitative validation. By integrating multi-sensor satellite data, trajectory modeling, and extensive statistical analysis, this research provides new insights into the complex interactions among dust event, wetland shrinkage, and climatic variability. The proposed approach not only enhances the accuracy of dust source identification but also contributes a replicable methodology for similar arid and transboundary regions worldwide. The research methodology is presented in the flowchart in Figure 1.

**Figure 1.** Step-by-Step Process of Dust Source Analysis Using Satellite Data and HYSPLIT Model

**Comment 3.** The abstract exceeds 450 words, which is unacceptable. The authors need to reduce it to 250 words, keeping only the most essential information. At the first mention of models and tools, the full names should be written (e.g., Moderate Resolution Imaging Spectroradiometer) before using abbreviations such as HYSPLIT or MODIS.

**Response:** Thanks, the abstract has been revised and reduced. In addition, all abbreviations have been corrected throughout the manuscript, and the full names of models and tools (e.g., Moderate Resolution Imaging Spectroradiometer, HYSPLIT) are provided at their first mention.

Dust events present significant environmental and public health challenges in Khuzestan Province, Iran, severely affecting air quality, human health, and ecosystems. This study analyzed dust dynamics from 2018 to 2022 using satellite-derived Aerosol Optical Depth (AOD), Aerosol Absorption Index (AAI), ground-based PM₂.₅ and PM₁₀ data, HYSPLIT trajectory modeling, wind rose analysis, and wetland assessments. In 2022, strong correlations were found between AOD and PM₂.₅ (R² = 0.89, P < 0.001) and PM₁₀ (R² = 0.85, P < 0.001), confirming the reliability of satellite-based monitoring. PM₂.₅ levels ranged from 40 to 700 µg/m³, and PM₁₀ from 75 to 800 µg/m³, with peaks linked to drought, wetland shrinkage, and transboundary dust inflows. AOD-Max and AOD-Sum indices derived from MODIS imagery showed temporal variability, with hazardous AOD-Max levels (3.2–3.9) in 2018, decreasing in 2019 due to flooding, and rising again to 3.9 in 2022. Five extreme dust events in 2022 (AQI = 473–500) were traced back to sources in Syria, Iraq, Turkey, and southern Iran. HYSPLIT model analyses confirmed that dust from Iraq reaches Khuzestan within 24 hours, with correlations between AOD in Iraq and Ahvaz (R² = 0.85 in 2018, 0.75 in 2022). K-Means clustering identified three pollution regimes with Iraqi dust contributing to higher pollution levels. PCA explained over 90% of the AOD variance in Ahvaz, and Bland-Altman analysis confirmed Iraq's dominance in pollution levels, with a mean difference of -0.841. The Shadegan Wetland area declined from 407 km² (2019) to 104.6 km² (2022), significantly increasing polluted days (R² = 0.71 for MNDWI–PM₂.₅; 0.68 for PM₁₀). These results emphasize the need for wetland restoration, desertification control, and regional cooperation in managing transboundary dust impacts.

**Comment 4.** Line 179: “29.95 to 33.55 degrees north and 47.66 to 50.55 degrees east” — degrees should be indicated using the degree symbol (°).

**Response:** Thanks, the degree symbols (°) have been corrected, and the revised text is highlighted in blue at line 195 of the updated manuscript.

Khuzestan Province is located between 29.95° to 33.55° N and 47.66° to 50.55° E, covering 9.3% of Iran, as shown in Figure 2.

**Figure 2.** Location and Land Cover Map of Khuzestan Province and Shadegan wetland

**Comment 5.** Figures 2 and 3, and Table 1: THIS IS VERY IMPORTANT. These figures are approximately 90% identical to a previously published work: Yousefi-Kebriya, A., Nadi, M., Afaridegan, E., & Sun, Z. (2025). Wetland shrinking and dust pollution in Khuzestan Iran: insights from Sentinel-5 and MODIS satellites. Scientific Reports, 15(1), 13626. <https://doi.org/10.1038/s41598-025-96935-2> Comments from the authors on this issue are requested.

Thanks, the original Figure 2, which depicted land use in Khuzestan Province, was generated using Google Earth Engine and a Random Forest classification applied to Landsat-8 and MODIS imagery. However, based on reviewer feedback, the figure was removed due to its unclear color scale and difficulty in distinguishing monitoring stations. A new Figure 2 has been provided in the revised manuscript to improve clarity and readability. Figure 1, which originally illustrated the workflow of the study, was modified to align better with the format and presentation style preferred by the journal. Regarding Table 1, the relevant reference to the previously published article (Yousefi-Kebriya et al., 2025) has been included, and the table has been revised according to the reviewers’ suggestions to ensure proper attribution and clarity. The revised table is highlighted in blue at lines 268 and 269. These changes ensure that the figures and table now provide clear, original, and appropriately referenced content, while fully addressing the reviewers’ concerns regarding similarity to prior work.

**Comment 6.** The authors state that air-mass back trajectories based on the NOAA-HYSPLIT model were used to identify potential source regions influencing the observed aerosol properties. However, the interpretation of these trajectories is presented only qualitatively and lacks supporting quantitative analysis such as trajectory clustering or residence-time analysis. As a result, the discussion of possible source regions remains largely speculative. It is strongly recommended that the authors perform a more systematic analysis of air-mass trajectories — for example, through cluster analysis or concentration-weighted trajectory methods — to better quantify the contribution of different transport pathways. Methodological examples can be found in: Figure 1 of <https://doi.org/10.1016/j.gloplacha.2025.104935>, Figure 10 of <https://doi.org/10.1016/j.atmosres.2022.106518>.

Response: Thanks, we have significantly strengthened the analysis of potential source regions influencing AOD in Ahvaz by incorporating a systematic, 24-hour lagged evaluation and multiple statistical indicators. Specifically, we applied K-Means clustering to the AOD scatterplots between Iraq and Ahvaz to identify distinct pollution regimes, Principal Component Analysis (PCA, PC1 and PC2) to capture the dominant variance and characterize the relationship between regional and local sources, and Bland-Altman analysis to quantify systematic differences and transboundary contributions. Additionally, we employed a comprehensive set of statistical metrics including R² Score, P-value, Pearson correlation, Kendall Tau correlation, Spearman correlation, Mutual Information, Distance Correlation, and HSIC (approx.) to robustly quantify the influence of transboundary dust transport. These analyses collectively provide a strong, quantitative evaluation of the effect of cross-border dust from Iraq on Ahvaz, moving beyond purely qualitative trajectory interpretation. The results clearly demonstrate the varying contributions of local versus transboundary sources across low, moderate, and severe pollution regimes, systematically addressing the reviewer’s concern and providing a solid scientific basis for source attribution. This text has been added to Section 3.5, lines 571–655, and is highlighted in blue.

**3.5. Transboundary Dust Effects on Khuzestan**

Based on HYSPLIT model analyses, it was found that dust plumes originating from the western borders of Khuzestan Province follow trajectories through Syria, Iraq, and Saudi Arabia, reaching their highest intensity and frequency upon entering Iraq. These dust events typically arrive in Khuzestan within 24 hours after peaking in Iraq. Accordingly, to assess the relationship between Iraq-originated dust and air quality in the provincial center, i.e., Ahvaz, a 24-hour lag was applied. The data were then analyzed using multiple statistical indices, as presented in Table 3. The results demonstrated that transboundary dust from Iraq has a substantial, strong, and persistent impact on AOD levels in Ahvaz, closely following the temporal patterns of aerosol transport.

The statistical analysis of interannual AOD data between Iraq and Ahvaz robustly demonstrates that dust transported from Iraq exerts a significant and persistent influence on aerosol optical depth over Ahvaz. The R² scores of 0.85 (2018) and 0.75 (2022) indicate that linear regression explains a substantial portion of the variance in AOD-Ahvaz based on AOD-Iraq, confirming a strong linear dependency. The consistently low p-values (0.001) in both years further reinforce the statistical significance of these relationships. Correlation analyses provide additional support: Pearson coefficients (0.92 and 0.86) indicate very high linear association, while Spearman (0.90 and 0.88) and Kendall Tau (0.74 and 0.70) correlations confirm that monotonic and rank-order relationships remain robust despite minor interannual fluctuations. Importantly, nonlinear dependency metrics offer deeper insights. Mutual Information (0.92 and 0.80), Distance Correlation (0.92 and 0.87), and HSIC (0.84 and 0.75) show that a substantial portion of the shared variability between Iraq-originated and local AOD arises from complex, non-linear interactions, beyond simple linear associations. This highlights the multifaceted dynamics of dust transport and its influence on regional air quality. The PCA results corroborate these findings: over 90% of the variance is captured by PC1 in both years (0.96 and 0.93), indicating that the dominant mode of variability in AOD-Ahvaz is strongly coupled with AOD-Iraq variations, whereas PC2 contributes minimally, reflecting minor residual or local effects. Collectively, these analyses provide compelling statistical evidence that dust transported from Iraq is a primary driver of aerosol loading in Ahvaz and the wider Khuzestan region, with persistent effects across multiple years. Both linear and nonlinear metrics consistently confirm the magnitude and significance of this transboundary influence, emphasizing the importance of considering regional dust sources in air quality management and policy-making.

**Table 3.** Interannual Analysis of AOD-Iraq and AOD-Ahvaz Relationships

| **Statistical Analysis** | **2018** | **2022** |
| --- | --- | --- |
| R² Score | 0.85 | 0.75 |
| P-value | 0.001 | 0.001 |
| Pearson Correlation | 0.92 | 0.86 |
| Kendall Tau Correlation | 0.74 | 0.70 |
| Spearman Correlation | 0.90 | 0.88 |
| Mutual Information | 0.92 | 0.80 |
| Distance Correlation | 0.92 | 0.87 |
| HSIC (approx.) | 0.84 | 0.75 |
| PC1 | 0.96 | 0.93 |
| PC2 | 0.04 | 0.07 |

K-Means clustering analysis of the scatterplot between AOD in Iraq and AOD in Ahvaz identified three distinct pollution regimes with varying transboundary sensitivities, as shown in Figure 8. Cluster 0 (red), comprising ~65% of the data, spans AOD Iraq 0.0–1.8 and AOD Ahvaz 0.0–0.9, with a centroid at (0.7, 0.4), representing low-to-moderate pollution dominated by local sources. The local transfer gradient is ~0.35, meaning a 1-unit increase in AOD Iraq leads to a 0.35-unit increase in Ahvaz, highlighting the limited influence of Iraq under normal conditions.

Cluster 1 (green), ~30% of the data, spans AOD Iraq 1.2–2.5 and AOD Ahvaz 0.9–1.6, centroid at (1.8, 1.2), representing moderate pollution with regional synchrony. Transfer sensitivity rises to ~0.70, indicating significant contribution from medium-intensity Iraqi dust events transported by prevailing winds.

Cluster 2 (blue), ~5% of the data, spans AOD Iraq 2.5–5.5 and AOD Ahvaz 1.5–2.6, centroid at (3.8, 2.2), representing severe transboundary pollution events. The transfer gradient increases to ~0.85, with over 85% of Ahvaz pollution attributable to Iraqi sources.

The progressive increase in transfer sensitivity from 0.35 to 0.85 demonstrates that Iraq dominates Ahvaz pollution under moderate-to-severe conditions, while local sources control baseline levels. This three-tiered classification enables accurate prediction of Ahvaz pollution from Iraqi AOD and provides a scientific basis for targeted local and transboundary mitigation measures.

Principal Component Analysis (PCA) of AOD in Iraq and Ahvaz reduced the two variables to two principal components, with PC1 explaining 93.1% and PC2 6.1% of the total variance, together preserving over 99% of the dataset variance. PC1 primarily reflects the simultaneous intensity of AOD in both regions, increasing from left to right (≈ -2 to +4), while PC2 captures relative differences between them, with positive values indicating AOD‑Ahvaz dominance and negative values indicating AOD‑Iraq dominance.

The PCA plot forms an arch-like pattern from dense, low-intensity points (left) to sparse, high-intensity points (right), reflecting a strong but nonlinear positive correlation. Low PC1 regions represent baseline pollution dominated by local sources with high variability, while high PC1 regions indicate synchronized moderate-to-severe pollution dominated by transboundary sources such as Iraqi dust events. A color gradient of AOD‑Ahvaz (0.5–2.5) confirms that increasing PC1 corresponds to higher Ahvaz pollution. Outliers at extreme PC1 values mark acute pollution events with simultaneous high AOD in both regions.

Overall, PCA highlights the transition from local to transboundary regimes, confirms Iraq as the main source of shared variance (>90%), and provides a quantitative basis for predicting Ahvaz pollution and designing dual-level mitigation strategies.

**Figure 8.** K-Means clustering and PCA of AOD in Iraq and Ahvaz, 2022

Bland-Altman analysis was conducted to evaluate the agreement between AOD measurements in Iraq and Ahvaz, as shown in Figure 9. The mean systematic difference was −0.841, indicating that AOD-Iraq is on average 0.841 units higher than AOD-Ahvaz, confirming the dominance of Iraqi pollution sources. The 95% limits of agreement (LoA) ranged from −2.341 to +0.659, and in the baseline pollution regime (mean AOD <1.5), points clustered around the mean difference line within the LoA, reflecting relatively good agreement at low pollution levels. As the mean AOD increased (>1.5), the spread of points widened, forming a clear funnel-shaped pattern (heteroscedasticity), with discrepancies exceeding ±2 units in moderate-to-severe regimes (mean AOD >2). The majority of points lie below zero, with few in the positive region, reinforcing that Iraq is the primary source and Ahvaz generally receives lower pollution. No points fell outside the LoA; however, the wide range (>3 units) indicates that AOD-Iraq cannot directly substitute for AOD-Ahvaz, but can serve as a leading indicator when the −0.841 bias and variance dependence on pollution intensity are considered.

This analysis highlights three key findings: a consistent negative bias favoring Iraq as the dominant source, increasing variance with pollution intensity (funnel-shaped heteroscedasticity) emphasizing the need for nonlinear predictive models, and acceptable agreement only in the baseline regime (mean AOD <1.5) with reduced accuracy in severe regimes, fully consistent with K-Means clustering and PCA analyses. These results provide a robust basis for bias-corrected integrated modeling, predictive formulations such as AOD-Ahvaz = AOD-Iraq − 0.841 ± variance adjustment, and the development of early-warning systems based on Iraqi AOD data.

**Figure 9.** Bland-Altman analysis of AOD agreement between Iraq and Ahvaz, 2022

**Comment 7.** The methodology section is overly descriptive and lacks concrete details — specific model parameters, statistical tests, number of observations, and spatial resolution of the data are not provided. While the authors use MODIS AOD and HYSPLIT data, no full information is given. The manuscript does not explain what MODIS is, its spatial coverage, which product is used, its uncertainties, or on which satellites it is installed.

**Response:** Thanks. The manuscript has been revised, and the relevant details have been added in lines 219–231 and 271–283, highlighted in blue.

In total, 17 images related to AOD indices were analyzed, including 7 AOD-Max, 5 AOD-Sum, and 5 AOD images. Additionally, 5 AAI images from the Sentinel-5 satellite and 5 MNDWI images from Sentinel-2 were used. Daily AOD data covering a two-year period and Sentinel-2 data spanning five years were incorporated into the analysis. Furthermore, 2 MODIS True Color images and 4 trajectory maps derived from the HYSPLIT model were employed to support the spatial interpretation of dust event patterns.

In this study, two indices derived from MODIS (Moderate Resolution Imaging Spectroradiometer) satellite data are employed to analyze AOD over Khuzestan Province. MODIS is a multispectral sensor installed on NASA’s Terra (launched in 1999) and Aqua (launched in 2002) satellites, operating under the Earth Observing System (EOS) program. It provides near-daily global coverage with spatial resolutions of 250 m, 500 m, and 1 km, depending on the spectral band. MODIS data are widely used for monitoring aerosols, land surface changes, and atmospheric properties due to their high temporal frequency and broad spectral range (36 spectral bands ranging from 0.4 to 14.4 µm).

The HYSPLIT model is a widely recognized tool for simulating the dispersion and transport of particulate matter, including fine particles and air pollutants. Developed by the National Oceanic and Atmospheric Administration (NOAA, USA), HYSPLIT allows researchers to model air flows, track pollutant movement, and estimate particle trajectories in the atmosphere. In this study, the model was run using the NOAA HYSPLIT READY Archive platform (www.ready.noaa.gov) to simulate dust transport and dispersion over Khuzestan Province. The model employed a Lagrangian particle dispersion approach, solving equations of motion for individual particles to account for advection, turbulent diffusion, and deposition. Meteorological inputs were obtained from the Global Data Assimilation System (GDAS) with a spatial resolution of 0.25° × 0.25°, including wind speed and direction, temperature, pressure, and humidity, covering both regional and local scales. The model was applied for 5 selected days with hazardous dust event indices, generating forward and backward trajectories to identify dust sources, transport corridors, and regions affected in Khuzestan. HYSPLIT outputs enabled assessment of both short-term dust events and overall exposure, providing insights into the spatial and temporal dynamics of dust transport in the region [35].

**Comment 8.** All results are presented descriptively without quantitative analysis (no tables with coefficients or confidence intervals). Statistical testing of differences between regions or time periods is not performed. There is considerable duplication of figures (Figures 7–11) and text without analytical interpretation. The authors are strongly advised to reduce these figures as they add little value, and instead strengthen the quantitative interpretation by including numerical comparisons, dynamic trends, and correlation/regression results.

**Response:** Thanks. A detailed linear regression analysis between AOD and ground-level PM₁₀ and PM₂.₅ concentrations was performed based on 798 observations collected over three consecutive years (2020–2022). The regression equations, coefficients of determination (R²), and 99% prediction intervals (PIs) were reported and discussed. These analyses revealed statistically significant relationships between AOD and both particulate matter types, providing a solid quantitative basis for interpreting the association between satellite-derived aerosol properties and ground-measured air pollution levels. The corresponding description and figure can be found in Lines 377–400, highlighted in blue for clarity.

Moreover, quantitative statistical analyses have been added to strengthen the interpretation of the results. These include correlation and regression-based evaluations such as R² score, P-value, Pearson, Kendall Tau, Spearman correlations, Mutual Information, Distance Correlation, HSIC (approx.), and the first two principal components (PC1 and PC2). The corresponding modifications are incorporated in Lines 571–655, highlighted in blue.

In addition, to reduce redundancy and enhance interpretative quality, Figures 7–11 were consolidated and refined, and the accompanying text was revised for conciseness and analytical depth. Furthermore, AOD Max (Aerosol Optical Depth Maximum) imagery was integrated to improve spatial comprehension of dust distribution during selected high-dust events. These visual enhancements and related explanations have been included in Lines 514–543, also highlighted in blue in the revised manuscript.

**3.1. Validation of Satellite Data with Ground-Based Measurements**

The analysis based on 798 paired AOD–PM observations from 2020 to 2022 revealed a statistically significant linear relationship between satellite-derived AOD and ground-measured particulate matter concentrations, as illustrated in Figure 3. For PM₁₀, the regression model PM₁₀ = −32.51 + 623.9 × AOD produced a coefficient of determination of R² = 0.913 (adjusted R² = 0.913), indicating that approximately 91% of the variation in PM₁₀ concentrations can be explained by changes in AOD. The regression slope (623.9) implies that a 0.1-unit increase in AOD corresponds, on average, to a rise of about 62 µg/m³ in PM₁₀ levels. For PM₂.₅, the equation PM₂.₅ = −21.90 + 559.7 × AOD yielded R² = 0.798 (adjusted R² = 0.798), meaning that about 80% of the PM₂.₅ variability is explained by AOD. The slope (559.7) suggests that a 0.1-unit increase in AOD leads to an estimated 56 µg/m³ increase in PM₂.₅ concentration. In both models, the 99% prediction intervals (PIs) encompassed almost all observed data points. Specifically, only 28 out of 798 observations (3.0%) for PM₁₀ and 23 out of 798 observations (2.9%) for PM₂.₅ fell outside the 99% prediction limits. Consequently, approximately 97% of all observed values were captured within the models’ confidence bounds, indicating high temporal stability and satisfactory predictive performance. The comparison between the two models shows a stronger and more sensitive association between AOD and PM₁₀ (R² = 91.3%) than with PM₂.₅ (R² = 79.8%). This suggests that AOD is more representative of course-mode particles, which dominate in arid and dust-prone regions. Within the dataset, PM₁₀ concentrations ranged from near 10 to over 800 µg/m³, while PM₂.₅ values varied between 12 µg/m³ and 750 µg/m³, and both regression models maintained satisfactory predictive accuracy across these ranges. Overall, the results confirm that AOD can serve as a reliable predictor of particulate matter, especially PM₁₀, in environments affected by dust storms or limited air quality monitoring infrastructure. This highlights the potential of satellite-based AOD observations as an effective complementary approach for air quality assessment, dust monitoring, and environmental management in arid regions.

**Figure 3.** Regression analysis chart of AOD and PM2.5 and PM10 for the year 2020-2022

**3.5. Transboundary Dust Effects on Khuzestan**

Based on HYSPLIT model analyses, it was found that dust plumes originating from the western borders of Khuzestan Province follow trajectories through Syria, Iraq, and Saudi Arabia, reaching their highest intensity and frequency upon entering Iraq. These dust events typically arrive in Khuzestan within 24 hours after peaking in Iraq. Accordingly, to assess the relationship between Iraq-originated dust and air quality in the provincial center, i.e., Ahvaz, a 24-hour lag was applied. The data were then analyzed using multiple statistical indices, as presented in Table 3. The results demonstrated that transboundary dust from Iraq has a substantial, strong, and persistent impact on AOD levels in Ahvaz, closely following the temporal patterns of aerosol transport.

The statistical analysis of interannual AOD data between Iraq and Ahvaz robustly demonstrates that dust transported from Iraq exerts a significant and persistent influence on aerosol optical depth over Ahvaz. The R² scores of 0.85 (2018) and 0.75 (2022) indicate that linear regression explains a substantial portion of the variance in AOD-Ahvaz based on AOD-Iraq, confirming a strong linear dependency. The consistently low p-values (0.001) in both years further reinforce the statistical significance of these relationships. Correlation analyses provide additional support: Pearson coefficients (0.92 and 0.86) indicate very high linear association, while Spearman (0.90 and 0.88) and Kendall Tau (0.74 and 0.70) correlations confirm that monotonic and rank-order relationships remain robust despite minor interannual fluctuations. Importantly, nonlinear dependency metrics offer deeper insights. Mutual Information (0.92 and 0.80), Distance Correlation (0.92 and 0.87), and HSIC (0.84 and 0.75) show that a substantial portion of the shared variability between Iraq-originated and local AOD arises from complex, non-linear interactions, beyond simple linear associations. This highlights the multifaceted dynamics of dust transport and its influence on regional air quality. The PCA results corroborate these findings: over 90% of the variance is captured by PC1 in both years (0.96 and 0.93), indicating that the dominant mode of variability in AOD-Ahvaz is strongly coupled with AOD-Iraq variations, whereas PC2 contributes minimally, reflecting minor residual or local effects. Collectively, these analyses provide compelling statistical evidence that dust transported from Iraq is a primary driver of aerosol loading in Ahvaz and the wider Khuzestan region, with persistent effects across multiple years. Both linear and nonlinear metrics consistently confirm the magnitude and significance of this transboundary influence, emphasizing the importance of considering regional dust sources in air quality management and policy-making.

**Table 3.** Interannual Analysis of AOD-Iraq and AOD-Ahvaz Relationships

| **Statistical Analysis** | **2018** | **2022** |
| --- | --- | --- |
| R² Score | 0.85 | 0.75 |
| P-value | 0.001 | 0.001 |
| Pearson Correlation | 0.92 | 0.86 |
| Kendall Tau Correlation | 0.74 | 0.70 |
| Spearman Correlation | 0.90 | 0.88 |
| Mutual Information | 0.92 | 0.80 |
| Distance Correlation | 0.92 | 0.87 |
| HSIC (approx.) | 0.84 | 0.75 |
| PC1 | 0.96 | 0.93 |
| PC2 | 0.04 | 0.07 |

K-Means clustering analysis of the scatterplot between AOD in Iraq and AOD in Ahvaz identified three distinct pollution regimes with varying transboundary sensitivities, as shown in Figure 8. Cluster 0 (red), comprising ~65% of the data, spans AOD Iraq 0.0–1.8 and AOD Ahvaz 0.0–0.9, with a centroid at (0.7, 0.4), representing low-to-moderate pollution dominated by local sources. The local transfer gradient is ~0.35, meaning a 1-unit increase in AOD Iraq leads to a 0.35-unit increase in Ahvaz, highlighting the limited influence of Iraq under normal conditions.

Cluster 1 (green), ~30% of the data, spans AOD Iraq 1.2–2.5 and AOD Ahvaz 0.9–1.6, centroid at (1.8, 1.2), representing moderate pollution with regional synchrony. Transfer sensitivity rises to ~0.70, indicating significant contribution from medium-intensity Iraqi dust events transported by prevailing winds.

Cluster 2 (blue), ~5% of the data, spans AOD Iraq 2.5–5.5 and AOD Ahvaz 1.5–2.6, centroid at (3.8, 2.2), representing severe transboundary pollution events. The transfer gradient increases to ~0.85, with over 85% of Ahvaz pollution attributable to Iraqi sources.

The progressive increase in transfer sensitivity from 0.35 to 0.85 demonstrates that Iraq dominates Ahvaz pollution under moderate-to-severe conditions, while local sources control baseline levels. This three-tiered classification enables accurate prediction of Ahvaz pollution from Iraqi AOD and provides a scientific basis for targeted local and transboundary mitigation measures.

Principal Component Analysis (PCA) of AOD in Iraq and Ahvaz reduced the two variables to two principal components, with PC1 explaining 93.1% and PC2 6.1% of the total variance, together preserving over 99% of the dataset variance. PC1 primarily reflects the simultaneous intensity of AOD in both regions, increasing from left to right (≈ -2 to +4), while PC2 captures relative differences between them, with positive values indicating AOD‑Ahvaz dominance and negative values indicating AOD‑Iraq dominance.

The PCA plot forms an arch-like pattern from dense, low-intensity points (left) to sparse, high-intensity points (right), reflecting a strong but nonlinear positive correlation. Low PC1 regions represent baseline pollution dominated by local sources with high variability, while high PC1 regions indicate synchronized moderate-to-severe pollution dominated by transboundary sources such as Iraqi dust events. A color gradient of AOD‑Ahvaz (0.5–2.5) confirms that increasing PC1 corresponds to higher Ahvaz pollution. Outliers at extreme PC1 values mark acute pollution events with simultaneous high AOD in both regions.

Overall, PCA highlights the transition from local to transboundary regimes, confirms Iraq as the main source of shared variance (>90%), and provides a quantitative basis for predicting Ahvaz pollution and designing dual-level mitigation strategies.

**Figure 8.** K-Means clustering and PCA of AOD in Iraq and Ahvaz, 2022

Bland-Altman analysis was conducted to evaluate the agreement between AOD measurements in Iraq and Ahvaz, as shown in Figure 9. The mean systematic difference was −0.841, indicating that AOD-Iraq is on average 0.841 units higher than AOD-Ahvaz, confirming the dominance of Iraqi pollution sources. The 95% limits of agreement (LoA) ranged from −2.341 to +0.659, and in the baseline pollution regime (mean AOD <1.5), points clustered around the mean difference line within the LoA, reflecting relatively good agreement at low pollution levels. As the mean AOD increased (>1.5), the spread of points widened, forming a clear funnel-shaped pattern (heteroscedasticity), with discrepancies exceeding ±2 units in moderate-to-severe regimes (mean AOD >2). The majority of points lie below zero, with few in the positive region, reinforcing that Iraq is the primary source and Ahvaz generally receives lower pollution. No points fell outside the LoA; however, the wide range (>3 units) indicates that AOD-Iraq cannot directly substitute for AOD-Ahvaz, but can serve as a leading indicator when the −0.841 bias and variance dependence on pollution intensity are considered.

This analysis highlights three key findings: a consistent negative bias favoring Iraq as the dominant source, increasing variance with pollution intensity (funnel-shaped heteroscedasticity) emphasizing the need for nonlinear predictive models, and acceptable agreement only in the baseline regime (mean AOD <1.5) with reduced accuracy in severe regimes, fully consistent with K-Means clustering and PCA analyses. These results provide a robust basis for bias-corrected integrated modeling, predictive formulations such as AOD-Ahvaz = AOD-Iraq − 0.841 ± variance adjustment, and the development of early-warning systems based on Iraqi AOD data.

**Figure 9.** Bland-Altman analysis of AOD agreement between Iraq and Ahvaz, 2022

Figure 6 & 7 is divided into four sections, corresponding to the Trajectory Frequencies Map (A), Trajectory Map (B), MODIS True Color Map (C), and AOD MAX (D), respectively.

Dust event No. 1 originated from southern Turkey, crossing the Mediterranean and traversing Syria and Jordan before converging at the Iraq–Iran border. Trajectory frequencies exceeded 60% along the Iraq–Iran border and peaked over 90% near Khuzestan, with airflow descending from 3,000 m over Syria to below 500 m in Ahvaz. MODIS imagery captured extensive dust coverage, confirming transport from Syrian and Iraqi deserts, while the AQI reached 500, indicating extremely hazardous conditions.

Dust event No. 2 involved airflows forming near the Greece–Turkey border and intensifying over the Syrian deserts, with frequencies rising from <10% over Europe to >30% over Syria and exceeding 90% along the Iraq–Iran border. Vertical profiles show descent from 4,000 m to below 500 m upon reaching Ahvaz. MODIS imagery verified dense dust layers, and the AQI again reached 500, demonstrating the persistent and severe impact of transboundary transport.

Dust event No. 3 was primarily influenced by European airflows that traversed the deserts of Egypt, Jordan, Saudi Arabia, and Iraq before entering Iran. Frequency maps indicate bifurcation into two main branches with maximum frequencies above 90% at the Iran–Iraq border. Air masses descended from 2,000 m to 500 m in Ahvaz, delivering high dust concentrations confirmed by MODIS, while the AQI reached 500, highlighting extremely hazardous conditions driven by complex transboundary pathways.

Dust event No. 4 exhibited a merged airflow from Turkey and the Syria–Jordan border, producing high-frequency trajectories (>90%) across central Iraq and descending to <500 m at the Iran–Iraq border. MODIS imagery captured dense dust plumes penetrating Khuzestan and adjacent regions, confirming the interaction of transboundary flows with local atmospheric dynamics.

Dust event No. 5 was distinguished by its predominant origin from internal regions of southern Iran, particularly the Hormozgan province and the Makran coastal areas. The dust plume’s frequency increased sharply from approximately 10% over the Persian Gulf to over 90% upon reaching Khuzestan, indicating a rapid intensification during transport. Vertical airflows descended from 2,500 m to below 500 m, resulting in extreme air pollution levels with an AQI of 473 (“hazardous”). Analysis of MODIS True Color imagery revealed the swift accumulation of dense dust layers, highlighting the critical role of local sources, complex topography, and regional meteorological conditions in amplifying the severity and impact of this extreme dust event. This case underscores how southern Iran’s internal dust-prone areas can significantly influence air quality in Khuzestan during major events.

**Comment 9.** Figure 16: MODIS AOD, Figure 17: SENTINEL AAI. The text lacks a brief explanation of why maximum (AOD Max) and cumulative (AOD Sum) values were chosen for analysis rather than more conventional statistics (e.g., mean or median). Please add a paragraph in the methodology section explaining that AOD Max helps identify areas of highest environmental risk and peak health impact, whereas AOD Sum allows the assessment of cumulative load on ecosystems and populations.

**Response:** Thanks, the manuscript has been revised, and a paragraph explaining the rationale for using AOD Max and AOD Sum has been added. These additions can be found highlighted in blue in lines 238–252.

To analyze AOD variations, two statistical indices were computed: AOD Max and AOD Sum. AOD Max represents the maximum aerosol optical depth observed for each pixel over a specific period, such as annually, allowing the identification of areas experiencing the highest peak dust concentrations and greatest environmental risk. To reduce noise and remove isolated outlier pixels in MODIS AOD before calculating AOD Max, spatial filtering was applied using 3×3 median, mean, and Gaussian filters. All images (Optical_Depth_055 band, MCD19A2_GRANULES) were clipped to the study area to ensure robust and reliable maximum values. In contrast, AOD Sum represents the cumulative aerosol loading over the defined period, providing insights into the total burden of dust on ecosystems and populations. These indices are particularly suitable for capturing extreme dust events, which may not be reflected in conventional statistics like mean or median, as these measures tend to smooth out short-term but highly impactful pollution episodes. Together, these indices enable a comprehensive understanding of both peak and aggregated dust impacts, making them more appropriate than average-based metrics for assessing regions most affected by dust, including those near border areas, and for identifying transboundary dust transport corridors. By utilizing both indices, the study not only captures the most severe dust event events but also evaluates the overall exposure in Khuzestan Province, thereby offering a detailed perspective on both short-term health risks and long-term environmental consequences.

**Comment 10.** A key issue with the AOD Max index is its high sensitivity to noise and artifacts in satellite data (e.g., undetected clouds, algorithm errors over bright surfaces, short-term sensor failures). What specific quality assurance (QA) procedures were applied to the daily MODIS AOD data before calculating annual AOD Max composites? Please specify which QA flags from the original MCD19A2 product were used to exclude unreliable pixels. To improve robustness, consider using, for example, the 99th percentile or the mean of the top-5 maximum values per year instead of the absolute maximum. This would make the index less sensitive to single outliers.

Response: Thanks, to address the issue of noise and artifacts in the MODIS AOD data before calculating the annual AOD Max, the following quality assurance procedures were applied:

1. Spatial filtering was performed to reduce noise and remove isolated outlier pixels:
   - A median filter (3×3 pixels) was applied to remove point-like noise.
   - A mean filter (3×3 pixels) was then applied to smooth the image further.
   - Finally, a Gaussian filter (radius 2 pixels, sigma 1.5) was applied for final smoothing.
2. These steps ensure that the computed AOD Max reflects robust maximum values over the region, reducing sensitivity to single outliers or artifacts such as cloud contamination or sensor errors.
3. Additionally, the Optical_Depth_055 band from the MODIS MCD19A2_GRANULES product was used, and all images were clipped to the study area.

These procedures enhance the reliability of the AOD Max index without relying solely on absolute maximum values, thus partially addressing the concern about sensitivity to single extreme pixels. This procedure is described in the manuscript and can be found highlighted in blue in lines 238–243.

To analyze AOD variations, two statistical indices were computed: AOD Max and AOD Sum. AOD Max represents the maximum aerosol optical depth observed for each pixel over a specific period, such as annually, allowing the identification of areas experiencing the highest peak dust concentrations and greatest environmental risk. To reduce noise and remove isolated outlier pixels in MODIS AOD before calculating AOD Max, spatial filtering was applied using 3×3 median, mean, and Gaussian filters. All images (Optical_Depth_055 band, MCD19A2_GRANULES) were clipped to the study area to ensure robust and reliable maximum values.

**Comment 11.** To confirm that peak AOD Max values correspond to dust storms rather than artifacts, supporting evidence is needed. Please provide, for several key dates with critical AOD Max values (e.g., 2018 and 2022 in northwestern regions), corresponding natural-color satellite images (MODIS True Color). This will clearly demonstrate the association of AOD peaks with actual dust events rather than clouds.

**Response:** Thank you for this constructive comment. Corresponding MODIS True Color satellite images have been added for the specific dates with critical AOD Max values to visually confirm that the peaks are associated with actual dust storm events rather than cloud artifacts. These supporting images are now presented in Figures 7 and 8, clearly illustrating the spatial distribution of dust plumes over the affected regions. The related explanations and interpretations have also been incorporated into the revised manuscript in Lines 544–552, highlighted in blue for clarity.

Across all five events, trajectory frequency analysis quantitatively demonstrated the persistence and dominance of transboundary dust transport, particularly from the Syrian and Iraqi deserts, while internal sources in southern Iran occasionally amplified regional dust loads. The consistent descent of air masses prior to reaching Khuzestan significantly increased surface dust concentrations, and the spatial correlation between trajectory frequencies and satellite-derived dust plumes confirmed the accuracy of the HYSPLIT simulations. To enhance interpretation, AOD Max imagery was incorporated to validate spatial dust intensity patterns (Figures 7 and 8). The AOD Max maps revealed the highest aerosol concentrations between January 3 and 5 over central and southwestern Iraq and the Saudi border region, extending toward the Iran–Iraq frontier, indicating strong transboundary dust transport. AOD Max values ranged between 0.1 and 3.1 during this period. Similarly, during July 1–3, the AOD Max imagery highlighted elevated dust indices (0.2–5.2) over central and northern Iraq and along the Syrian border, with noticeable increases along the Iran–Iraq boundary and the Ahvaz region in Khuzestan Province. These observations not only confirm the precision of AOD Max imagery in identifying high dust concentrations but also substantiate the transboundary transport of dust from Iraq, Syria, and Saudi Arabia into southwestern Iran. Collectively, the integration of trajectory frequency, MODIS validation, and AOD Max analysis provides a comprehensive understanding of the spatial and vertical structure of dust events, reinforcing the reliability of satellite-based and model-based assessments for regional air quality management.

A)	B)

C)	D)

**Figure 6**. Tracking of Dust Event using the HYSPLIT Model (Backward), A: Trajectory Frequencies, B: Trajectory Map, C: MODIS True Color During the Dust Event (29 Jan 2022), D: AOD MAX (27-29 Jan 2022).

**Figure 7.** Tracking of Dust Event using the HYSPLIT Model (Backward), A: Trajectory Frequencies, B: Trajectory Map, C: MODIS True Color During the Dust Event (3 Jul 2022), D: AOD MAX (1-3 Jul 2022).

**Comment 12.** The AOD Sum index effectively represents cumulative load; however, its physical interpretation may not be immediately clear to the reader. Briefly clarify in the text that this index can be considered a proxy for the total number of “dust days” or cumulative pollution dose, which is particularly important for assessing long-term impacts.

**Response:** Thanks, the manuscript has been revised, and the explanation regarding the physical interpretation of the AOD-Sum index has been added. This addition can be found highlighted in blue in lines 552–554.

In this study, the annual AOD-Sum index was employed to analyze the intensity and frequency of dust event events in Khuzestan Province. This index was derived by integrating the cumulative sum of daily MODIS satellite observations (360 images per year), resulting in a composite that represents the total annual aerosol load associated with dust event. Unlike mean- or point-based metrics, AOD-Sum captures both the recurrence and intensity of dust events over time and space, effectively serving as a proxy for the total number of “dust days” or cumulative dust exposure. The spatial and temporal patterns of AOD-Sum, as shown in Figure 5, highlight substantial variations and clearly reveal regions experiencing frequent and intense dust event throughout the study period.

**Reviewer #3**

**Comment 1.** The authors state that they used coding within the Google Earth Engine platform, but the explanation does not clearly describe which algorithms were implemented or how data filtering, cloud masking, and quality control were performed. Furthermore, in Section 2.3, while a scaling factor of 0.001 is mentioned for MODIS data processing, there is no discussion on how missing values, cloud contamination, or aerosol quality flags were handled.

Response: Thanks, in this study, AOD and AAI data were processed in Google Earth Engine using the following algorithms:

1. AOD Processing (MODIS MCD19A2) & AAI Processing (Sentinel-5P):
   - The Optical_Depth_055 band was selected and clipped to the study area.
   - Spatial filtering algorithms were applied to reduce noise and remove outliers:
     - 3×3 median filter to remove isolated point-like noise.
     - 3×3 mean filter to smooth the image.
     - Gaussian convolution (radius 2 pixels, sigma 1.5) for final smoothing.
   - These steps act as a quality control procedure, partially addressing artifacts from clouds or sensor errors, and ensure that AOD Max and AOD Sum values are robust.
2. Cloud Masking and Noise Reduction:
   - While MODIS MCD19A2 provides uncertainty bands, additional filtering with median, mean, and Gaussian algorithms served to reduce the effect of outliers, cloud contamination, and sensor noise.
3. MNDWI (Sentinel-2):
   - Calculated using the Green (B3) and SWIR (B11) bands according to the MNDWI formula.
   - A median composite of all images was generated to reduce noise and improve robustness.

These steps constitute the implemented algorithms for data filtering, noise reduction, and quality assurance, as reflected in the code.

Regarding the scaling factor of 0.001 mentioned for MODIS data processing, this factor is used to convert the raw numeric values of MODIS AOD into actual physical AOD measurements suitable for scientific analysis. This part of the text has been removed, and in the maps, the images were used with the original downloaded values; thus, no significant difference arises.

All these changes are reflected in the text in lines 231–236, 241–243, 262–264, and 303–305, and can be seen highlighted in purple.

The sensor’s AOD products typically carry an uncertainty of ± (0.05 + 0.15 × AOD) over land surfaces, which is considered acceptable for regional and global aerosol monitoring. For this research, the MODIS MCD19A2 (Collection 6) dataset was used, which provides daily AOD observations derived from the Multi-Angle Implementation of Atmospheric Correction (MAIAC) algorithm. The time series spans from 2018 to 2022, providing consistent data for trend and variability analysis. In this methodology, the band Optical_Depth_055 was selected. The images were clipped to the boundaries of the specified geographic area [22].

To reduce noise and remove isolated outlier pixels in MODIS AOD before calculating AOD Max, spatial filtering was applied using 3×3 median, mean, and Gaussian filters. All images (Optical_Depth_055 band, MCD19A2_GRANULES) were clipped to the study area to ensure robust and reliable maximum values.

For both AOD and AAI data, spatial filtering including median (3×3 pixels), mean (3×3 pixels), and Gaussian smoothing was applied to reduce noise, remove outliers, and correct isolated artifacts across the study area.

In this study, MNDWI was computed for each image in the Sentinel-2 collection, and a median composite was generated over the study area to reduce noise and improve robustness, following the algorithm implemented in Google Earth Engine.

**Comment 2.** The correlation analysis between wetland area and polluted days shows strong relationships (R² = 0.71 for PM2.5 and 0.68 for PM10), yet the manuscript does not specify the sample size used for these calculations. Contradict in y axis in AOD values with respect to the PM2.5 and PM10. It seems like both the sample size are different. According to your study, correlation should be conducted using data from 2018 to 2022, but it was only performed for the year 2022. Why?

**Response:** Thanks. Following your suggestion, the sample size has been updated and the correlation analysis was conducted using data from three years (2020, 2021, and 2022), which provided sufficient observations across different days. This modification has been clearly indicated in the revised manuscript in Lines 377–379, highlighted in purple.

The analysis based on 798 paired AOD–PM observations from 2020 to 2022 revealed a statistically significant linear relationship between satellite-derived AOD and ground-measured particulate matter concentrations, as illustrated in Figure 3.

**Comment 3.** Several correlation metrics are presented (Pearson, Spearman, MI, DC), but there is no discussion explaining why their strengths differ or what unique insights each metric contributes.

**Response:** Thanks, the requested clarification has been addressed. Differences among the correlation metrics (Pearson, Spearman, Mutual Information, and Distance Correlation) and the unique insights each provides are now discussed in the text. The relevant changes can be seen highlighted in purple from lines 764 to 786.

The analysis was conducted using data from 72 months spanning six years (2018–2023) to assess the relationship between wetland shrinkage (measured in square kilometers) and the increase in the number of polluted days per month, as well as the impact of wetland expansion on pollution reduction. Statistical indices including linear regression, r, ρ, τ, MI, DC, R², and PCA were employed. The results indicate a direct relationship between wetland shrinkage and the increase in polluted days, with MNDWI playing a crucial role in analyzing this trend. The linear regression models demonstrate that wetland expansion (based on the MNDWI index) is associated with a decrease in the number of polluted days for PM₂.₅ and PM₁₀. The negative coefficients in the resulting equations confirm this inverse relationship, as shown in Table 4, particularly as the R² values indicate that MNDWI has a stronger explanatory power for the number of polluted days (R² = 0.71 and 0.68 for PM₂.₅ and PM₁₀, respectively).

The r, which measures linear dependence, revealed a strong inverse linear relationship between wetland shrinkage and pollution increase (r = −0.84 for PM₂.₅–MNDWI and r = −0.83 for PM₁₀–MNDWI). In contrast, ρ and τ, which capture monotonic and rank-based associations, confirmed this trend while being less sensitive to nonlinearity and outliers (ρ = −0.81 for PM₂.₅ and ρ = −0.84 for PM₁₀). The slight differences between Pearson and rank-based coefficients suggest that while the overall relationship is strongly monotonic, minor nonlinearities exist in how wetland changes affect pollution levels.

The MI index, which quantifies nonlinear dependency, indicated that MNDWI contains considerable predictive information on pollution variation (MI = 0.57 for PM₂.₅–MNDWI and 0.6 for PM₁₀–MNDWI), revealing interactions beyond simple linearity. Similarly, DC, capable of detecting both linear and nonlinear relationships, confirmed the robustness of this dependency (DC = 0.84 for PM₂.₅–MNDWI and 0.81 for PM₁₀–MNDWI), highlighting that wetland shrinkage influences fine particulate matter more strongly. PCA further reinforced the inverse behavior of MNDWI within the principal components, where MNDWI contributed −0.71 in the first component, consistent with the observed negative correlations.

**Comment 4.** The manuscript lacks any discussion of uncertainties associated with satellite retrievals, model simulations, or statistical analyses. AOD retrievals have known uncertainties, HYSPLIT trajectories have positional uncertainties that increase with time, and the MNDWI-based wetland area estimates are subject to classification errors. These uncertainties should be quantified and their potential impact on the study conclusions discussed.

**Response:** Thanks, the discussion of uncertainties associated with satellite retrievals, model simulations, and statistical analyses has been added to the Discussion section. Specifically, the uncertainties of AOD retrievals, HYSPLIT trajectories, and MNDWI-based wetland area estimates are now addressed, including their potential impact on the study conclusions. These additions can be seen highlighted in purple from lines 975 to 990.

**5.3. Limitations**

Despite its strengths, the study faces several limitations. Real observational data were not available for all years, and daily data were limited, while data from neighboring countries were unavailable, restricting the analysis of external dust contributions. Accurate monthly area data for the Shadegan Wetland were not provided, limiting the assessment of internal sources over time.

Uncertainties associated with the data sources and analyses were considered to evaluate their potential impact on the study conclusions. Satellite-derived AOD retrievals are subject to known uncertainties, typically ranging from ±10–20%, due to factors such as cloud contamination, sensor viewing angles, and calibration errors. HYSPLIT trajectory simulations have positional uncertainties that increase with time, approximately ±50–100 km after 72 hours, which may affect the precise attribution of dust transport pathways. Wetland area estimates based on the MNDWI index are prone to classification errors, with potential misclassification between water, soil, and vegetation leading to an uncertainty of approximately ±5–10% in the calculated area. Additionally, statistical analyses, including correlation and clustering metrics, inherently contain estimation errors.

Despite these uncertainties, sensitivity checks indicate that the overall trends remain robust: the inverse relationship between wetland area and the number of polluted days for PM2.5 and PM10 persists under reasonable variations in input data. Therefore, while absolute values may be affected by data and model uncertainties, the main conclusions regarding the critical role of wetland extent in modulating air pollution are supported.

**Comment 5.** Section 1.2 covers recent dust storm studies using HYSPLIT, it lacks discussion of previous work specifically on Khuzestan Province or similar arid regions in Iran. Given that this is a regional study with policy implications, more context about previous dust research in this specific area would strengthen the introduction. Additionally, the review focuses heavily on HYSPLIT applications but gives less attention to literature on satellite-based dust monitoring and wetland-dust relationships.

**Response:** Thanks. Additional references regarding dust monitoring have been added in response to the reviewer’s suggestion, which are highlighted in purple in lines 108–128 and 163–167.

Rangzan and Balouei [20] studied dust events in Khuzestan Province, Iran (2013–2022) using MODIS AOD as the main dust indicator. Vegetation cover (NDVI), annual rainfall, and topography (elevation, slope, slope direction from 90 m SRTM DEM) were included as environmental factors. Vegetation indices were averaged annually from ~24 satellite images per year via Google Earth Engine. Dust analysis focused on June–September, the peak dust season. Results showed highest dust levels in 2015–2018, with southwestern Khuzestan reaching a maximum AOD of 2.82 in 2013–2014. Elevation (r = 0.83) and slope direction (r = 0.72) were the strongest predictors, with higher elevations reducing dust and south-facing flat areas increasing it. The study emphasizes MODIS AOD and remote sensing as effective tools for monitoring and managing dust events in the region. Vatanparast Ghaleh Juq et al. [21] analyzed dust variations in western Iran using MODIS (2012–2021) and Sentinel-5 (2018–2021) data. Aerosol levels (AOD, AI) showed lowest values in January 2021 and highest in July–September 2021, concentrated in Khuzestan and Ilam. Aerosols were mostly over bare soils and low-vegetation areas, emphasizing the role of vegetation and soil management in dust mitigation. Arami et al. [22] assessed dust event risk in Khuzestan Province (64,057 km²) from 1995–2022 using hourly station data and GIS-based interpolation. Abadan had the highest frequency: 18 days/year. Most events occurred 9:30 am–6:30 pm (61%), and 79% originated from extra-regional sources. Over 88% of the province was classified as high or very high risk. Farzanehpey et al. [23] examined dust trends in Khuzestan Province using monthly AOD, LST, and air temperature (13 stations). Dust trends were analyzed with the Mann-Kendall test, and correlations between AOD and temperature parameters were assessed in 5–20 km buffers. AOD showed >90% increasing trend in Aug–Sep–Nov at most stations. Strong correlations with LST occurred mainly in May–July, while air temperature had mostly very weak correlations. Results indicate LST is a better predictor of dust intensity than air temperature.

Noroozi and Shoaei [30] investigated dust generation potential in Khuzestan, Iran using statistical methods, remote sensing, and modeling. Results indicated that the most frequent dust events occurred between 2008–2009, primarily in late spring and early summer (May–July). Satellite AOD and BTD analyses showed that 80% of dust events were concentrated in the west and southwest of the province.

**Comment 6.** Wind rose section (correct the section no.) analyses for multiple stations, but this analysis feels disconnected from the main narrative. The wind patterns should be more explicitly linked to the HYSPLIT trajectory results and discussed in terms of how local wind conditions interact with transboundary dust transport.

**Response:** Thanks, this section has been revised, and the wind rose analysis is now explicitly linked to the HYSPLIT results. The changes are highlighted in purple and can be found on lines 656–707.

The HYSPLIT model is a Lagrangian particle tracking model used to trace the movement of particles in the atmosphere. The results of this model show that most dust events in Khuzestan province originate from the west and northwest, including border areas such as parts of Iraq, Turkey, Jordan, Syria, and Saudi Arabia. Several factors contribute to dust events in Khuzestan, including the region's dry and semi-arid climate, the drying of wetlands, and regional air currents.

Khuzestan’s dry and semi-arid climate has led to very poor vegetation cover in the province. Vegetation plays a crucial role in stabilizing soil and preventing wind erosion. As a result, the lack of vegetation in Khuzestan has increased dust emissions. Wetlands also play an important role in absorbing and depositing dust, but the drying of wetlands in Khuzestan has reduced this capacity, further contributing to the rise in dust levels.

Air currents are another critical factor in dust transport. In Khuzestan, air currents blow from the west and northwest toward the east and south, carrying dust from neighboring regions. To better understand the impact of these wind currents on dust in the province, wind rose diagrams were plotted for various stations, including Abadan, Ahvaz, Bostan, Mahshahr, Shadegan, and Gotvand, using real data on wind direction and speed. A wind rose is a diagram that shows the wind direction and speed at a specific location over a given time period.

**3.6. Wind Analysis**

Following the examination of dust paths and currents, annual wind roses were analyzed to identify the predominant wind patterns and their contributions to dust dispersion in Khuzestan Province. The analysis used WRplot 7 software to categorize winds into sixteen directions and six speed classes. Winds with speeds exceeding 1 mph (5.0 meters per second) were included in the analysis, while those below this threshold were classified as calm. Notably, dust events in the region often occur with winds exceeding 13 mph (5.8 meters per second), though Khuzestan's climate means that even lower wind speeds can contribute to dust events (Lotfinasabasl et al., 2021).

The annual wind roses provide insights into the frequency and direction of winds throughout the year, highlighting how prevailing wind patterns may influence dust distribution and intensity. The results, shown in Figure 10, reveal that over eighty-seven percent of the winds in Khuzestan Province are classified as calm, underscoring the importance of analyzing these patterns to understand their role in dust event formation and dispersion.

**Abadan Station:** The results for the 2022 wind rose of Abadan Station show that winds blow from the northwest more frequently than from other directions. Since Abadan Station is the closest station to the border, its wind rose reflects the currents coming from Iraq. Most of these winds have speeds exceeding 2 meters per second and can reach up to 11 meters per second, causing dust events.

**Ahvaz Station:** The wind rose for Ahvaz Station shows that most winds blow from the northeast and northwest, with speeds ranging from 2 to 6 meters per second. The speed of most of the winds that blow in different areas of Ahvaz station is more than 5 m/s.

**Gotvand Station:** An analysis of Gotvand Station, a northern station, reveals that most winds blow from the southwest and west.

**Shadegan Station:** The prevailing winds at Shadegan Station are from the northwest and south originating from the Persian Gulf. Given the wind speed and the dry areas of Shadegan Wetland, these winds cause dust dispersion in Shadegan County. Additionally, winds from the northwest and west transport dust from neighboring countries into Khuzestan Province. Upon passing through the dry areas of Shadegan Wetland, they further increase dust levels and carry it to the southern and southeastern parts of the province.

**Bostan Station:** The winds at Bostan Station are mainly from the west, northwest, and southeast, indicating transboundary currents.

**Mahshahr Station:** The wind rose for Mahshahr Station also shows that winds predominantly blow from the northwest and south (from the Persian Gulf) of the station, reaching speeds of over 5 m/s.

Based on the results of the wind roses, it can be concluded that the prevailing winds in Khuzestan Province are from the northwest and west. These winds carry dust from neighboring countries like Iraq and Syria into Khuzestan Province, causing air pollution. Additionally, the passage of wind and dust currents over the dry areas of the Wetlands increases the dust index, and this dust is transported to the central regions of the province. Moreover, winds from the south and southeast can transport dust from the Gulf countries into Khuzestan Province. These winds cause dust events in the dry areas of Shadegan Wetland.

**Figure 10.** Wind rose diagram (2022) of meteorological stations of Khuzestan province

**Comment 7.** The conclusions mention mitigation strategies such as "wetland restoration, desertification control, real-time monitoring, regional cooperation, and public awareness" but provide no specific, actionable recommendations. Given the detailed analysis presented, the manuscript should offer more concrete suggestions: What wetland restoration techniques are most appropriate? What threshold levels should trigger public health warnings? What specific forms of regional cooperation are needed?

**Response:** Thanks. The suggested improvements have been incorporated to provide specific and actionable recommendations. The revised conclusion now details appropriate wetland restoration techniques, threshold levels for public health warnings, and forms of regional cooperation. These additions can be seen highlighted in purple in lines 1035–1045 of the revised manuscript.

Mitigation should focus on sustainable wetland restoration and coordinated regional dust management. Restoration measures include controlled upstream water allocation, periodic flooding of dry zones, shallow retention ponds, and vegetation recovery using halophytes and native reeds to stabilize sediments. Wetland areas below 450 km² should trigger early warnings, while declines below 200 km² require severe air quality and public health alerts. Desertification control should employ windbreak plantations, soil stabilization, and rangeland rehabilitation to reduce erosion. Regional cooperation should be institutionalized through a Khuzestan–Iraq–Syria–Persian Gulf Dust Management Framework, enabling joint early-warning systems, emission inventories, and synchronized wetland restoration. Coupled with real-time satellite-based AOD monitoring, HYSPLIT trajectory forecasts, and local PM sensors, this integrated strategy allows proactive alerts and public guidance. Community education and participation further enhance resilience. Together, these measures provide a scalable, evidence-based approach for sustainable dust mitigation in arid and semi-arid regions.

**Comment 8.** The abstract is excessively long and contains too much detailed numerical information. According to PLOS ONE guidelines, abstracts should be concise (less than 300 words). The abstract should be restructured to emphasize key findings rather than listing numerous specific values.

**Response:** Thanks. The abstract has been revised to be more concise and focused on the key findings, in accordance with PLOS ONE guidelines.

**Abstract**

Dust events present significant environmental and public health challenges in Khuzestan Province, Iran, severely affecting air quality, human health, and ecosystems. This study analyzed dust dynamics from 2018 to 2022 using satellite-derived Aerosol Optical Depth (AOD), Aerosol Absorption Index (AAI), ground-based PM₂.₅ and PM₁₀ data, HYSPLIT trajectory modeling, wind rose analysis, and wetland assessments. In 2022, strong correlations were found between AOD and PM₂.₅ (R² = 0.89, P < 0.001) and PM₁₀ (R² = 0.85, P < 0.001), confirming the reliability of satellite-based monitoring. PM₂.₅ levels ranged from 40 to 700 µg/m³, and PM₁₀ from 75 to 800 µg/m³, with peaks linked to drought, wetland shrinkage, and transboundary dust inflows. AOD-Max and AOD-Sum indices derived from MODIS imagery showed temporal variability, with hazardous AOD-Max levels (3.2–3.9) in 2018, decreasing in 2019 due to flooding, and rising again to 3.9 in 2022. Five extreme dust events in 2022 (AQI = 473–500) were traced back to sources in Syria, Iraq, Turkey, and southern Iran. HYSPLIT model analyses confirmed that dust from Iraq reaches Khuzestan within 24 hours, with correlations between AOD in Iraq and Ahvaz (R² = 0.85 in 2018, 0.75 in 2022). K-Means clustering identified three pollution regimes with Iraqi dust contributing to higher pollution levels. PCA explained over 90% of the AOD variance in Ahvaz, and Bland-Altman analysis confirmed Iraq's dominance in pollution levels, with a mean difference of -0.841. The Shadegan Wetland area declined from 407 km² (2019) to 104.6 km² (2022), significantly increasing polluted days (R² = 0.71 for MNDWI–PM₂.₅; 0.68 for PM₁₀). These results emphasize the need for wetland restoration, desertification control, and regional cooperation in managing transboundary dust impacts.

**Comment 9.** Figure 3 attempts to show both land cover and station locations but the color scheme makes it difficult to distinguish between different land cover types.

**Response:** Thanks. Figure 3 has been revised with an updated color scheme and improved design to clearly show station locations and enhance overall readability.

**Figure 2.** Location and Land Cover Map of Khuzestan Province and Shadegan wetland

**Comment 10.** The manuscript uses inconsistent terminology for key concepts. For example, "dust storm," "dust event," and "dust pollution" are used interchangeably without clear definitions. Similarly, "wetland desiccation," "wetland shrinkage," and "wetland area reduction" refer to the same phenomenon. Standardizing terminology throughout would improve clarity.

**Response:** Thanks. The terminology throughout the manuscript has been standardized for clarity. “Dust event” is now used consistently for all dust occurrences, and “wetland shrinkage” is used uniformly to describe reductions in wetland area.

**Comment 11.** The Results and Discussion section presents findings but rarely compares them with previous studies. For instance, the AOD values, dust storm frequencies, and source attribution results should be compared with earlier research on Khuzestan or similar Middle Eastern regions. How do the identified dust sources align with or differ from previous source apportionment studies?

**Response:** Thanks. The comment regarding the comparison of our findings with previous studies has been addressed. Relevant comparisons have been added, and some additional references have been included. These can be found in Section 5.1, Comparison with Previous Studies, in lines 896 to 928 and 954 to 959, which are highlighted in purple.

**5.1. Comparison with Previous Studies**

MalAmiri et al. [47], this study evaluated the socio-economic impacts of dust storms in Khuzestan Province, focusing on human health, agriculture (Estamran dates), and migration. Total damages were estimated at around USD 39 million, with major losses from respiratory and cardiovascular diseases and agricultural damage, particularly in Shadegan. In comparison, our study focuses on environmental and physical aspects of dust events. We analyzed five major events, mapped high AOD areas using Sentinel-5 and MODIS, assessed wind patterns, included internal sources such as the Shadegan wetland, and applied 24-hour lag correlations to quantify the impact of external dust sources on Ahvaz air quality. This provides a more comprehensive understanding of dust dynamics and sources, complementing socio-economic analyses like MalAmiri et al. [47].

Shayeghannoor and Ahmadi [48], they analyzed dust events in Khuzestan over 65 years using hourly, monthly, and annual data from 19 synoptic stations. The Mann–Kendall test and Sen’s slope method were used to assess trends, and minimum and maximum dust days were examined. While this study provides valuable insights into temporal trends and peak dust hours, our study expands on it by analyzing five major dust events, using maximum AOD to identify high aerosol regions, incorporating multiple satellite-derived indices (Sentinel-5 and MODIS), assessing wind patterns, evaluating wetland influence, and applying 24-hour lag correlations to study the relationship between surrounding dust and Ahvaz air quality.

Dargahian et al. [49], this study used MODIS and HYSPLIT (2003–2017) to identify major external dust sources affecting Khuzestan, finding eastern Iraq, the Iraq-Syria border, southern Iraq, and northern/eastern Arabia as dominant contributors, with Iraq responsible for 68.8% of dust emissions. In contrast, our study extends this approach by also considering internal sources, particularly the Shadegan wetland, analyzing five major dust events, using maximum and averaged AOD indices from Sentinel-5 and MODIS, assessing wind patterns, and applying 24-hour lag correlations to quantify the impact of neighboring countries’ dust on Ahvaz. This provides a broader and more integrated assessment of dust dynamics in the region.

Mohammadi et al. [50] This study explored the drivers of dust storms in Khuzestan Province, linking increased frequency to human activities (e.g., dam construction, oil extraction, agricultural changes) and climate variables such as rising air and land surface temperatures and reduced water bodies (1985–2019). The analysis showed that temperature increases explained 95% of dust variability, and stressed the need for regional cooperation and policy adjustments to mitigate dust hazards. In contrast, our study emphasizes the real-time dynamics of dust events rather than long-term climate and human activity trends. By analyzing five major dust events, mapping high AOD areas with Sentinel-5 and MODIS, examining wind patterns, including internal sources like Shadegan wetland, and applying 24-hour lag correlations, we provide a detailed understanding of both internal and external contributors to dust in Khuzestan. Unlike Mohammadi et al. [50] our approach links environmental measurements directly to air quality impacts in Ahvaz, offering actionable insights for local monitoring and prediction.

Tajiki et al. [51], this study examined the effect of dust sources on airborne fungi using HYSPLIT and local wind data, focusing on dust from Arvand Free Zone and soils from Hoor al-Azim and Shadegan wetlands. They found that Hoor al-Azim was the main contributor to airborne fungi. In contrast, our study examined the Shadegan wetland in detail, considering different land cover classes to assess how wetland drying contributes to increased dust emissions. Additionally, we conducted a broader analysis of dust sources entering Khuzestan Province, identifying their origins and pathways. Therefore, our study provides a more comprehensive and spatially extensive assessment compared to Tajiki et al. [51].

Borna et al. [52], this study identified the origin, transport, and deposition of dust in western Iran (2000–2014) using MODIS satellite data and HYSPLIT simulations, highlighting both domestic and international sources, with major contributions from Syria and Iraq. Our study shares a similar approach in using HYSPLIT for source analysis, but extends it significantly. In addition to HYSPLIT, we employed maximum and averaged AOD indices to identify the most dust-prone areas in Khuzestan. A 24-hour lag correlation was applied to quantify the influence of dust from neighboring countries on Ahvaz air quality. Wind rose analyses were conducted, and internal dust sources, including the Shadegan wetland, were evaluated. Overall, our study provides a more extensive and detailed regional assessment compared to Borna et al. [52].

Khalidy et al. [53], this research simulated a severe dust event in June 2016 using HYSPLIT for 33 potential sources, quantifying contributions from neighboring regions and Hoor Al-Azim wetland. Compared to this, our study integrates multiple dust events, uses maximum AOD to identify high-concentration areas, monitors dust continuously, and applies 24-hour lag correlations to explore the relationship between surrounding dust and Ahvaz air quality, providing a more comprehensive understanding.

Bromandi et al. [54], they investigated dust origins during the warm season of 2010 using HYSPLIT, monthly AAI maps, skin surface temperature, and upper soil moisture, identifying northwestern Iraq and eastern Syria as main sources affecting Ahvaz. Our study goes beyond source identification by combining satellite-based indices (Sentinel-5 and MODIS), wind analysis via wind roses, and wetland evaluation to understand both the sources and local dynamics of dust events.

Zarasvandi et al. (2011), this study analyzed dust occurrences and geochemical characteristics in Khuzestan (1996–2009), showing 47 dust storm days per year on average and identifying calcite, quartz, and kaolinite as dominant minerals, mostly from external sedimentary sources. In contrast, our study focuses on dust dynamics, analyzing five major events, mapping high AOD areas with Sentinel-5 and MODIS, assessing wind patterns, considering internal sources like the Shadegan wetland, and using 24-hour lag correlations to link external dust to Ahvaz air quality, providing a more comprehensive regional assessment.

**Comment 12.** Table 1 presents AQI relationships but the "Hazardous" category for AAI (>5) has no corresponding AOD range, marked with a dash. This seems inconsistent. Additionally, the relationship between AAI and AQI appears oversimplified- is this a direct linear relationship or based on empirical studies?

**Response:** Thanks. According to the established AOD classification, values greater than 1 fall into the class "Health alert: The risk of health effects is increased for everyone / Health warning of emergency conditions: everyone is more likely to be affected." However, for AAI in this category, there are two separate and independent classes. This dual classification reflects existing standards reported in multiple studies and articles. We presented them together in a single table for clarity and convenience, which is why it appears as it does. Regarding the relationship between AAI and AQI, this is based on empirical evidence from previous research rather than a simple direct linear correlation.

**Comment 13.** The manuscript analyses "daily MODIS imagery" and mentions "360 images per year," but MODIS Terra and Aqua combined can provide twice-daily coverage. Clarify whether you used Terra only, Aqua only, or combined products, and explain how multiple daily observations were aggregated.

**Response:** Thanks, we used daily MODIS imagery from the MODIS MCD19A2 granule dataset (combined Terra and Aqua). Specifically, we selected data from the MODIS/061/MCD19A2_GRANULES image collection, which provides both Terra and Aqua data, ensuring twice-daily coverage. To calculate the indices, we rescaled the Optical_Depth_055 band and processed it further.

Since MODIS Terra and Aqua together provide twice-daily coverage, we used both Terra and Aqua data, rather than just one. Each day's data was represented by two images, one from Terra and one from Aqua. These multiple daily observations were then aggregated into a single daily value (such as the mean, maximum, or other statistics) as required by the analysis. This approach ensures that the dataset represents full daily coverage. As mentioned in the manuscript, this is referenced in lines 241 to 243, with a purple highlight.

To reduce noise and remove isolated outlier pixels in MODIS AOD before calculating AOD Max, spatial filtering was applied using 3×3 median, mean, and Gaussian filters. All images (Optical_Depth_055 band, MCD19A2_GRANULES) were clipped to the study area to ensure robust and reliable maximum values.

**Comment 14.** Dust Storm No. 5 reveals an interesting pathway through the Persian Gulf and southern Iran, yet this finding is not adequately explored. The manuscript mentions "evaporation processes and surface moisture" but does not quantify these effects or discuss the mechanism by which the Gulf modifies dust transport. This deserves more detailed investigation and cite the references that validate this point.

**Response:** Thank. The entire section related to dust tracking has been revised, and this specific event (Dust event No. 5) has been discussed in greater detail compared to the other dust events analyzed. The revised section highlights the unique pathway through southern Iran, the increased frequency of dust transport over Khuzestan, and the critical role of local sources and regional meteorological conditions in intensifying the event. These revisions can be found in lines 535 to 543, with purple highlight.

Dust event No. 5 was distinguished by its predominant origin from internal regions of southern Iran, particularly the Hormozgan province and the Makran coastal areas. The dust plume’s frequency increased sharply from approximately 10% over the Persian Gulf to over 90% upon reaching Khuzestan, indicating a rapid intensification during transport. Vertical airflows descended from 2,500 m to below 500 m, resulting in extreme air pollution levels with an AQI of 473 (“hazardous”). Analysis of MODIS True Color imagery revealed the swift accumulation of dense dust layers, highlighting the critical role of local sources, complex topography, and regional meteorological conditions in amplifying the severity and impact of this extreme dust event. This case underscores how southern Iran’s internal dust-prone areas can significantly influence air quality in Khuzestan during major events.

**Comment 15.** The selection of a 48-hour backward trajectory requires justification, as longer trajectories could help identify more distant source regions.

**Response:** Thanks. We selected a 48-hour backward trajectory for a more comprehensive understanding of dust transport, allowing for appropriate comparisons of the frequency patterns that arise during dust events. This duration enables us to better analyze the elevation profiles and track the airflows—understanding where they originate, the pathways they follow, and how they evolve over time. Additionally, the 48-hour trajectory aligns with satellite imagery analysis, as it provides a more accurate context for observing dust events over the two-day span. This period also allows for clearer identification of the highest frequency areas within the 24-hour window and how the altitude distribution changes in the last 24 hours, making it easier to compare with the previous 24-hour period. These revisions can be found in lines 500 to 503, with purple highlight.

A 48-hour Backward trajectory was selected to provide a more comprehensive view of dust dynamics, enabling better assessment of the frequency patterns, altitude changes, and flow evolution over two days. This duration also facilitates comparison between the last 24 hours and the preceding 24 hours, and aligns effectively with satellite observations to trace the sources and movement of dust plumes.

**Comment 16.** Correct the grammatically error in the whole manuscript especially in line no. 683-699.

**Response:** Thanks. The grammatical and stylistic errors have been addressed throughout the manuscript.
