# Supplementary material for: Analysis and source identification of dust event in Khuzestan province using MODIS and SENTINEL satellite data and HYSPLIT model (2018–2022)
Source: PLoS One. 2026 May 7;21(5):e0348129. doi: 10.1371/journal.pone.0348129 (PMC13152170; doi:10.1371/journal.pone.0348129)
Supplement: Attachment — Submitted filename: Minor comments.docx [file pone.0348129.s007.docx]

**Comments to Author:**

Author addressed most of the comments successfully in the revised manuscript entitled “Analysis and Source Identification of Dust event in Khuzestan Province Using MODIS and SENTINEL Satellite Data and HYSPLIT Model (2018-2022)” carefully. Still some minor changes need to be corrected before acceptance of the manuscript.

**Comment 1:**

In line no. 259-260: AOD definitions confuse standard concepts: AOD is misdefined as "measured in terms of the number of air particulate molecules per cubic kilometer". (AOD is dimensionless, representing optical extinction). Correct the sentence it.

**Comment 2:**

Throughout the manuscript, PM2.5 and PM10 are mentioned at some places and PM_2.5_ PM_10_ at others. Please use these symbols uniformly in the whole manuscript.

**Comment 3:**

In line numbers 215, 251, 450, 483: correct the word dust ‘event’ to ‘events’.
