# Supplementary material for: Analysis and source identification of dust event in Khuzestan province using MODIS and SENTINEL satellite data and HYSPLIT model (2018–2022)
Source: PLoS One. 2026 May 7;21(5):e0348129. doi: 10.1371/journal.pone.0348129 (PMC13152170; doi:10.1371/journal.pone.0348129)
Supplement: Attachment — Submitted filename: Response_to_reviewers_auresp_2.docx [file pone.0348129.s008.docx]

**Authors' Response to the Reviewers' Comments**

**Submission ID:** PONE-D-25-51186R1

**Title**: Analysis and Source Identification of Dust event in Khuzestan Province Using MODIS and SENTINEL Satellite Data and HYSPLIT Model (2018–2022)

Dear Editor,

Thank you for providing us the opportunity to revise and resubmit our manuscript, *“Analysis and Source Identification of Dust event in Khuzestan Province Using MODIS and SENTINEL Satellite Data and HYSPLIT Model (2018–2022)”*, for publication in your esteemed journal. We sincerely appreciate the time and effort you and the reviewers have invested in evaluating our work. Their detailed feedback and constructive suggestions have greatly enhanced the quality of our manuscript.

We have carefully reviewed and addressed all the reviewers’ comments and suggestions. Our responses are organized systematically, detailing the changes made to the manuscript to incorporate each suggestion. Below, we provide a comprehensive summary of our revisions and responses:

**Reviewer #2**

**Comment 1.** Despite the frequent mention of the HYSPLIT model, its application in the manuscript remains largely indirect and qualitative. The study does not employ standard quantitative trajectory-analysis techniques (trajectory clustering, PSCF, CWT, residence-time analysis), and the interpretation of dust sources is effectively replaced by correlation-based analysis between AOD in Iraq and Ahvaz. Such statistical dependencies demonstrate temporal coherence but do not provide physically grounded, mass-transport–based source attribution. As a result, the role of HYSPLIT is substantially weaker than claimed, and the conclusions regarding dominant source regions remain partially speculative. The authors are strongly encouraged to consult https://doi.org/10.1016/j.gloplacha.2025.104935 (Figure 1) and https://doi.org/10.1016/j.atmosres.2022.106518 (Figure 10) to refine the cluster-analysis methodology. These works should also be cited in the manuscript.

**Response:** We sincerely thank the reviewer for this valuable suggestion. Implementing the recommended improvements significantly enhanced our results, which could potentially lead to substantial advancements in dust management strategies in Khuzestan Province. The corresponding revisions have been highlighted in blue and are located from lines 610 to 682, as detailed below.

**3.4.1 Clustered Dust Pathways**

In this study, a total of 220 dust transport trajectories were analyzed for the year 2022, which was selected as a representative year characterized by high levels of dust pollution. These trajectories were generated using the HYSPLIT model driven by GLDAS meteorological data with a spatial resolution of 1° and a 3P configuration, and were computed as backward trajectories. Subsequently, a trajectory clustering procedure was applied to identify the dominant dust transport patterns. The results of this analysis are presented in Figure 8 and Table 3.

The backward trajectory analysis revealed six primary transport pathways with distinct contributions to dust loading over Ahvaz. Pathway 1, originating from Kuwait, accounted for 8.70% of the total trajectories and exhibited a relatively minor influence on the dust burden over the city. Pathway 2, which traverses the western border regions of Iran and eastern Iraq and passes over the desiccated areas of the Hoor Al-Azim Wetland, represented the dominant pathway, contributing 43.48% of the total trajectories and exerting the strongest influence on dust pollution in Khuzestan Province and Ahvaz. Pathway 3, encompassing the shared regions of southwestern Kuwait and southeastern Iraq, contributed 13.04% of the total. Pathway 4, originating from southern Iran and internal sources, particularly the provinces of Fars and Bushehr, accounted for 8.70% and showed a limited contribution to dust loading. Pathway 5, extending from the desert regions of Iraq and further influenced by source areas in Syria and Jordan, contributed a substantial 21.74% and played a significant role in enhancing the dust influx into Ahvaz. Finally, Pathway 6, associated with air mass flows originating from Europe and Turkey and subsequently passing over Syria and Iraq before reaching Ahvaz, accounted for only 4.35%, representing the least influential transport route.

The integrated assessment of these pathways indicates that, with the exception of Pathways 1 and 4, all remaining transport classes traverse the desert regions of Iraq, collectively accounting for 82.6% of the total dust transport routes. This pattern underscores the strategic and dominant role of Iraqi desert landscapes as primary source and transfer zones for dust affecting Khuzestan Province and the city of Ahvaz. Moreover, the high proportion of air mass flows passing through the western border regions of Iran and northeastern Iraq (exceeding 40%) highlights the potential for targeted management strategies, transboundary cooperation, and source-control policies in these areas to substantially mitigate the dust burden impacting western Iran, particularly Ahvaz, and to enhance the effectiveness of environmental and public health risk management.

**Table 3.** Percentage Share of Dust Trajectory Clusters

| Cluster | Ratio |
| --- | --- |
| 1 | 8.70% |
| 2 | 43.48% |
| 3 | 13.04% |
| 4 | 8.70% |
| 5 | 21.74% |
| 6 | 4.35% |

**Figure 8.** Backward Trajectory Clusters for Dust Transport

**3.4.2 Dust Emission Hotspots: WPSCF & WCWT Analysis**

The analysis of the WPSCF and WCWT maps presented in Figure 9 indicates that the main dust hotspots are located along a northwest–southeast belt in Iraq, aligning with the prevailing regional wind direction (northern Shamal winds) from Iraq and Syria toward Khuzestan. According to the WPSCF map, where AOD values range from 0 to 1, areas with values approaching 1 were identified as primary dust sources. Central, eastern, northern, and northeastern Iraq particularly regions surrounding the dried Hoor al-Azim wetland, the Mesopotamian basin, and abandoned agricultural lands around Baghdad with AOD values between 0.6 and 0.8, play the most significant role in dust particle generation. Additionally, the Syria–northwestern Iraq border, exhibiting high AOD indices, is recognized as a key transboundary source that directs dust particles along a southeastward path. Areas with moderate AOD values (0.4–0.6), including southern Iraq, Kuwait, and the border regions between Iraq and Khuzestan, act as secondary sources. These areas complete the transport pathways of dust plumes and contribute to increasing the concentration of suspended particles as they reach Khuzestan. The results indicate that during the studied days, particle concentrations in these regions were exceptionally high, with backward trajectories passing through these same points.

The WCWT index analysis further revealed that critical cores with AOD values greater than 1 are predominantly concentrated in two key regions: the central and southern Mesopotamian basin and the northwestern border strip of Khuzestan. In the Mesopotamian basin, the lowlands between the Tigris and Euphrates rivers particularly dried wetlands such as Hoor al-Hammar and the western parts of Hoor al-Azim have become major dust sources due to drought and reduced soil moisture. The northwestern border strip of Khuzestan, exhibiting high index values, indicates that dust particles generated in these cores enter the province with minimal deposition and maximum kinetic energy, intensifying local dust concentrations.

The elongated, linear arrangement of colored pixels from northwest to southeast reflects the dominance of stable synoptic flows, especially the northern Shamal winds. Areas with moderate AOD values (0.6–0.9) in the deserts of eastern Syria and Ninawa Province act as “amplifiers”; the passage of air masses through these regions gradually loads dust, reaching saturation points in central Iraq. In contrast, pixel discontinuities in remote northwestern areas suggest that contributions from distant deserts to severe events in Khuzestan are limited, as coarse particles tend to deposit over longer transport paths.

High AOD values near the borders of Khuzestan (0.9 to above 1) indicate that incoming air masses not only carry long-range dust but also interact with dense dust plumes generated in local cores, such as southern and eastern Ahvaz, leading to sudden increases in pollution indices at ground stations.

Overall, this analysis demonstrates that the dust crisis in Khuzestan operates within a transboundary system: distant sources in Syria and northern Iraq generate the initial cores of dust plumes, while the dried lands of southern Iraq and the Khuzestan border strip act as amplifying reservoirs, producing the highest AOD values at the closest geographical proximity to the province. These results clearly highlight the simultaneous role of transboundary and local sources in the occurrence of severe dust events and can serve as a basis for management policies aimed at mitigating the environmental and health impacts of this phenomenon.

**Figure 9.** Source Contribution and Dust Transport Pathways to Khuzestan Based on WPSCF and WCWT Models

**Comment 2.** The methodological section has been expanded and formally improved, yet in its current form it remains overloaded and conceptually unfocused. An excessive number of statistical methods is applied without clear justification for their necessity or hierarchy, and many of them ultimately confirm the same relationship between regional and local aerosol loading. This accumulation of techniques gives the impression of methodological inflation rather than a hypothesis-driven analytical framework. Reducing and structuring the methodological toolkit would considerably improve clarity and reproducibility.

**Response:** Thanks. The methodology has been revised, and the number of statistical indices has been reduced to focus on the main metrics, including R², Pearson Correlation, Spearman Rank Correlation, Mutual Information (MI), and K-Means Clustering. These indices were selected because each provides complementary insights: R² quantifies the proportion of variance explained, Pearson Correlation captures linear relationships, Spearman Rank Correlation identifies monotonic associations, Mutual Information (MI) reveals both linear and non-linear dependencies, and K-Means Clustering highlights natural groupings in the data. Other statistical metrics that largely confirmed the same relationships were removed to streamline the analysis and improve clarity. The corresponding revisions are highlighted in blue from lines 382 to 397. Moreover, the methodology section throughout the manuscript has been updated to reflect these changes, with all additions in the Materials and Methods section also highlighted in blue.

**2.9. Statistical Analysis**

To provide a clear framework for evaluating the relationships among the study variables, key statistical indices were selected to quantify linear and non-linear associations. These indices offer a rigorous basis for interpreting the dependencies and interactions present in the dataset.

**Coefficient of Determination (R²):** Coefficient of Determination (R²): Measures how well independent variables explain the variability of the dependent variable. Values near 1 indicate strong explanatory power, while lower values suggest other influencing factors [38].

**Pearson Correlation:** This coefficient measures the linear association between two variables, providing insight into their co-variation and direction of influence [39].

**Spearman Rank Correlation:** By ranking values, this non-parametric method captures monotonic relationships, making it robust for non-linear datasets [40].

**Mutual Information (MI):** MI measures shared information between variables based on entropy. Higher MI values indicate stronger dependencies, capturing both linear and non-linear interactions [43].

**K-Means Clustering:** This unsupervised approach partitions data into K clusters based on similarity. The algorithm iteratively assigns data points to centroids and recalculates their positions until convergence. Techniques such as the Elbow Method and Silhouette Score help identify the optimal number of clusters [45].

**Comment 3.** Figure 1 raises serious concerns regarding scientific graphics quality and the correct representation of the methodological workflow. Visually, it appears to be generated automatically or by AI, which is suggested by generic iconography, inconsistent typography, and incorrect scientific notation—most notably the suspicious rendering of “PM2.5” and “PM10.” The figure must be completely redrawn manually using proper scientific notation and a more rigorous, discipline-appropriate design.

**Response:** Thanks. Figure 1 has been revised and completely redrawn to ensure proper scientific notation and a rigorous, discipline-appropriate design.

**Figure 1.** Step-by-Step Process of Dust Source Analysis Using Satellite Data and HYSPLIT Model

**Comment 4.** Despite the revisions, the question of conceptual originality relative to the authors’ previously published article in Scientific Reports (2025) remains insufficiently resolved. Although the analysis period is extended and additional statistical metrics are introduced, the core region, datasets, physical interpretation, and key conclusions remain largely similar. The authors should clearly and explicitly articulate what fundamentally new scientific question this work addresses, beyond methodological elaboration of already published material.

**Response:** Thanks. The previous section has been completely removed and replaced with a new analysis. Based on HYSPLIT trajectory modeling and standard quantitative trajectory-analysis techniques (including trajectory clustering, PSCF, and CWT), it was determined that the Hoor al-Azim Wetland lies within the key dust-corridor entering Khuzestan Province. Therefore, the study examined monthly changes in the wetland’s surface area and water level (depth). For 2022, monthly maps of Hoor al-Azim were generated to provide detailed information on fully dried areas of the wetland. To assess its impact on dust events, the relationships between the wetland dynamics and dust were analyzed using R² and K-Means Clustering, highlighting the correlation with AOD and clarifying the wetland’s role in dust generation in the province. These revisions are highlighted in blue from lines 788 to 902, as detailed below.

**3.7. Assessment of Hoor Al-Azim Wetland Shrinkage**

The extensive wetlands of Khuzestan Province, particularly the Shadegan and Hoor al-Azim Wetlands, have historically acted as natural filters by trapping and depositing suspended particles, thereby reducing atmospheric dust levels and mitigating the intensity and frequency of local dust events. Under conditions of severe hydrological stress, however, this filtration capacity weakens, increasing the vulnerability of the system to internal dust generation. Based on recent assessments, 2022 was identified as the driest year for these wetlands within the 2018–2022 period (Yousefi Kebriya et al., 2025). Considering that more than 70% of dust transport trajectories pass through Hoor al-Azim Wetland, this study specifically examined the hydrological condition of the wetland (i.e., its water availability or dryness) and its relationship with dust events during 2022.

The variations in wetland surface water extent (derived from the MNDWI index) and the water level of the Hoor al-Azim Wetland over a five-year period (2018 to the end of 2022), as well as the relationship between these variables, are illustrated in Figure 12. During 2018, both indicators exhibit relatively low and stable values, with the surface area generally remaining below 900 km² and water levels fluctuating between approximately 1.7 and 2.9 m, reflecting a moderately constrained hydrological regime. A pronounced expansion phase is observed throughout 2019 and early 2020, characterized by a substantial increase in wetland surface area, reaching a peak of nearly 2,000 km² in April 2019, concurrently with elevated water levels exceeding 5 m. This synchronous response indicates a strong positive relationship between water availability and the spatial extent of surface water, confirming the sensitivity of the MNDWI-derived area to variations in wetland inundation depth. From mid-2020 onward, both variables display a gradual declining trend. The wetland surface area contracts steadily from values above 1,500 km² to below 1,000 km² by late 2021, while water levels decrease from approximately 4.0 m to around 2.1–2.5 m. This phase reflects a transition toward hydrological stress and partial desiccation of the wetland system. The most critical conditions are observed in 2022, during which the wetland experiences an abrupt and severe contraction. Surface area values derived from MNDWI drop dramatically, reaching a minimum of approximately 73 km² in July 2022, accompanied by a marked decline in water levels to nearly 1.2–2.9 m. This decoupling at very low water levels suggests extensive exposure of the wetland bed and fragmentation of surface water patches, indicating extreme drying conditions.

Overall, the relationship between variations in wetland surface area derived from MNDWI and changes in water depth (water level) is statistically significant, with a coefficient of determination of R² = 0.71, indicating a strong and meaningful linkage between spatial extent and hydrological conditions. The observed fluctuations are predominantly seasonal in nature; however, the five-year record reveals a pronounced long-term declining trend in both surface water area and water depth. This sustained reduction indicates progressive hydrological degradation of the wetland system, culminating in severe drying conditions in 2022, during which the Hoor al-Azim Wetland experienced a critical phase of water scarcity and extensive contraction of surface water coverage.

**Figure 12.** Temporal dynamics and regression-based relationship between Area (Km^2^) and water level in the Hoor al-Azim Wetland (2018–2022)

The spatiotemporal patterns of the MNDWI index for the twelve months of 2022 in the Hoor al-Azim Wetland are presented in Figure 13. The results indicate that the wetland was predominantly subjected to persistent dry conditions and a substantial reduction in surface water extent throughout the year. In January, MNDWI values ranged from 0.42 to −0.29, reflecting moderate to high dryness across large portions of the wetland, with surface water mainly confined to limited areas in the eastern, northwestern, and parts of the southeastern sectors, while the remaining regions exhibited characteristics of dry land. In February, the index range (0.43 to −0.28) showed no marked change compared to the previous month, indicating the persistence of stable but hydrologically constrained conditions. In March, a decline in MNDWI values to 0.35 to −0.25 was accompanied by a noticeable retreat of surface water in the eastern and southeastern sectors, suggesting an intensification of drying processes relative to earlier months. In April, although the index range remained broadly similar, the spatial distribution of surface water continued to be limited to scattered patches in the northwestern and eastern parts of the wetland, while extensive areas of the central and southern regions remained dry.

In May, despite a relative increase in MNDWI values to 0.38 to −0.27, the surface water extent further contracted, and the southeastern sector largely transitioned to dry conditions, leaving only restricted areas in the eastern and northwestern portions of the wetland with detectable surface water. This trend intensified during June and July. In July, with MNDWI values decreasing to 0.33 to −0.25, the wetland reached its annual minimum in surface water extent, and surface water was primarily limited to small, fragmented patches in the eastern sector and a minor area in the northwestern part, indicating a critical phase of wetland desiccation. In August, a slight and localized recovery of surface water was observed. In September, a relative increase in MNDWI values to 0.37 to −0.28 corresponded to a modest expansion of surface water in parts of the eastern and northwestern sectors. However, in October, November, and December, the index values remained within a similar range, and the spatial pattern indicated the stabilization of dry conditions across much of the wetland. During this period, the western, central, northern, and southern regions were predominantly characterized by persistent dryness, with surface water confined to limited areas in the eastern, northwestern, and parts of the southeastern sectors.

Overall, the results for 2022 demonstrate widespread and sustained desiccation of the Hoor al-Azim Wetland, which not only substantially reduced its hydrological and ecological capacity but also significantly weakened its natural function in trapping and depositing suspended particles. Under these conditions, the exposed and dried wetland surfaces become increasingly susceptible to acting as active sources of dust, while the wetland’s ability to attenuate and filter externally transported particulate matter is markedly diminished.

**Figure 13.** Monthly variations of the water surface area of the Hoor al-Azim Wetland based on the MNDWI in 2022

To examine the relationship between the reduction of the Hoor Al-Azim Wetland area and its impact on dust emissions in Khuzestan, a statistical analysis and K-Means clustering were performed on the wetland surface area and the AOD surrounding the wetland, as shown in Figure 13. The R² was 0.78, indicating a strong relationship in which variations in wetland area explain a significant portion of the changes in AOD. Figure 13 further illustrates three distinct clusters representing the wetland’s conditions, ranging from fully healthy to critical. The first cluster, indicated in purple at the lower right of the plot, represents an ideal and hydrologically healthy state, characterized by a large wetland area (approximately 1100–2000 km²) and very low AOD values (mostly below 0.4). These points correspond to wet periods, such as in 2019 and early 2020, when the extensive wetland acted as a natural filter, suppressing the suspension of particulate matter and maintaining air quality. The second cluster, shown in yellow at the center of the plot, represents a transitional and alert condition, with a moderate wetland area (400–1000 km²) and medium AOD values (0.4–1.0). This cluster indicates water stress; as the wetland area decreases, marginal dust sources become active and AOD begins to rise. Such conditions typically occur at the end of summer or during dry years, like 2018, signaling the onset of a potential crisis. The third cluster, depicted in green/blue at the upper left, represents a critical state and an active dust source, characterized by a very small wetland area (less than 250 km²) and very high AOD values (above 1.0, up to 1.7). These points reflect the most critical conditions, as observed in 2022, when the wetland was nearly dry, turning its bed into one of the most active internal sources of dust. A clear inverse relationship between wetland area and AOD is evident; as the area decreases, dust concentrations increase exponentially.

The cluster centroids, marked by red stars, indicate the average behavior of each cluster. The centroid of the critical cluster suggests that when the wetland area approaches approximately 150 km², AOD is expected to reach a hazardous level of 1.4, whereas the centroid of the healthy cluster indicates that maintaining clean air (AOD around 0.25) requires a wetland area above 1500 km². The staged reduction of wetland area is particularly noteworthy: the first reduction stage, from 1500 to 700 km², leads to a relative AOD increase of about 0.4 units, while the second stage, from 700 to 150 km², results in a sharp AOD rise of approximately 0.7 units, highlighting the exponential growth of dust concentration under critical conditions. The clustering analysis, as presented in Figure 13, clearly identifies a “red line” for wetland area at around 500 km², below which the wetland’s capacity to suppress dust emissions drastically decreases and the system enters a critical phase (AOD > 1). This information can be instrumental for dust crisis management in Khuzestan, enabling the identification of critical wetland areas. Overall, the combined cluster and regression analysis not only demonstrates the direct linkage between wetland hydrology and air quality but also establishes the minimum wetland area required to prevent substantial dust increases, providing a practical framework for environmental monitoring and management.

**Figure 13.** K-Means clustering of Hoor Al-Azim Wetland based on surface area and surrounding AOD

**Comment 5.** In several sections, correlation-based relationships are interpreted as causal (e.g., dominance of transboundary sources or the influence of wetland degradation on pollution levels). Without explicit causal modeling or physical validation, such interpretations should be substantially softened or clearly presented as associative rather than causal.

**Response:** Thanks. Following the reviewer’s suggestion to apply trajectory clustering, PSCF, and CWT, we incorporated these analyses prior to this section, which now provides a reasonable justification for the results. Nevertheless, the text has been further revised in accordance with the reviewer’s recommendations. These changes are highlighted in blue from lines 689 to 702.

**3.4.3 Transboundary Dust Effects on Khuzestan**

Based on HYSPLIT model analyses, it was found that dust plumes originating from the western borders of Khuzestan Province follow trajectories through Syria, Iraq, and Saudi Arabia, reaching their highest intensity and frequency upon entering Iraq. These dust events typically arrive in Khuzestan within 24 hours after peaking in Iraq. Accordingly, to assess the relationship between Iraq-originated dust and air quality in the provincial center, i.e., Ahvaz, a 24-hour lag was applied. The data were then analyzed using selected statistical indices, as presented in Table 3.

The results indicate a strong association between transboundary dust from Iraq and AOD levels in Ahvaz, reflecting temporal coherence with aerosol transport patterns. Linear regression analysis shows R² scores of 0.85 (2018) and 0.75 (2022), suggesting that a substantial portion of the variance in AOD-Ahvaz corresponds with AOD-Iraq. Consistently low p-values (0.001) in both years confirm the statistical significance of these relationships. Correlation analyses further support this association. Pearson coefficients (0.92 and 0.86) indicate high linear correlation, Spearman correlations (0.90 and 0.88) show robust monotonic relationships despite minor interannual fluctuations, and Mutual Information (0.92 and 0.80) reveals shared variability that includes nonlinear dependencies. Collectively, these analyses provide strong statistical evidence of a close temporal and quantitative association between dust levels in Iraq and aerosol loading in Ahvaz and the wider Khuzestan region. These results, supported by trajectory clustering, PSCF, and CWT analyses, indicate a clear association between regional dust transport and aerosol levels in Ahvaz, emphasizing the relevance of transboundary contributions in understanding and interpreting air quality patterns.

**Table 3.** Interannual Analysis of AOD-Iraq and AOD-Ahvaz Relationships

| **Statistical Analysis** | **2018** | **2022** |
| --- | --- | --- |
| R² Score | 0.85 | 0.75 |
| P-value | 0.001 | 0.001 |
| Pearson Correlation | 0.92 | 0.86 |
| Spearman Correlation | 0.90 | 0.88 |
| Mutual Information | 0.92 | 0.80 |

**Comment 6.** Despite the stated availability of code and data, the reproducibility of the analysis remains limited. The manuscript does not clearly specify HYSPLIT configuration parameters, the selection criteria for dust-event days, or the exact rules used to construct the AOD-Max and AOD-Sum indices. I fully understand that the authors used daily-resolution data from the official HYSPLIT archive and that higher temporal resolution is not available. However, this makes it even more important to follow my earlier recommendation to apply trajectory clustering according to the methodology outlined in the papers cited above.

**Response:** We sincerely thank the reviewer for this constructive and very detailed suggestion. The relevant section has been added, and trajectory clustering along with quantitative trajectory-analysis techniques such as PSCF and CWT have been incorporated into the manuscript, highlighted in blue from lines 609 to 981. This addition provides a clearer understanding of the application of the indices and the selection of dust-event days. The criteria for selecting dust-event days for the HYSPLIT web model focused on five days in 2022 with the highest dust indices (AQI = 500). Each of these days followed distinct trajectories, and together with trajectory clustering and quantitative particle-transport analyses (PSCF and CWT), they enabled a better understanding of dust transport pathways.

Additionally, AOD-Max and AOD-Sum indices were used to identify areas with the highest dust intensity and the greatest daily accumulation of dust. These analyses highlighted the border regions and the dry areas surrounding the Hoor al-Azim Wetland, supporting the manuscript’s further analyses by clarifying the reasons behind elevated AOD-Max and AOD-Sum values. This approach also contributed significantly to identifying the most polluted regions in Khuzestan Province, which is explicitly discussed in the manuscript.

**Comment 7.** Sections 2.8.1–2.8.5 should be removed or moved to the supplementary materials.

**Response:** Thanks. Sections 2.8.1–2.8.5 have been removed as suggested.

**Comment 8.** Several statistical metrics (PCA, Bland–Altman, MI, HSIC) are interpreted with excessive confidence, without discussion of their limitations or assumptions. In particular, the use of Bland–Altman for comparing regional and local AOD requires more careful justification, as these quantities are not alternative measurements of the same underlying variable in the strict methodological sense.

**Response:** Thanks. The number of statistical metrics has been reduced, and several indices have been removed to improve clarity and robustness. In particular, PCA, Bland–Altman, and HSIC were excluded in accordance with the reviewer’s suggestion, allowing the results to be presented more clearly and appropriately.

**Comment 9.** The authors are also strongly encouraged to cite more recent literature on dust storms, including:

<https://doi.org/10.1016/j.gsf.2023.101762>

<https://doi.org/10.1175/BAMS-D-23-0121.1>

<https://doi.org/10.1007/s11069-024-07003-3>

**Response: Response:** Thanks. All the recommended references have been incorporated into Section 1.2 (Review of Previous Studies) and are highlighted in blue from lines 95 to 126, as detailed below.

Filonchyk et al. (2025) investigated aerosol properties in Antarctica using AERONET data from seven stations and MERRA-2 reanalysis. The study analyzed aerosol optical depth (AOD), volume size distribution (0.09–0.76 μm), and direct aerosol radiative forcing (DARF). Results showed that aerosols are mainly natural (sea salt and marine sulfates), with AOD values ranging from 0.027 ± 0.019 to 0.082 ± 0.042, lowest at inland and highest at coastal sites. Surface radiative forcing indicated cooling effects (−14.3 ± 4 to −3 ± 0.5 W/m²), while atmospheric heating rates varied from 0.03 ± 0.005 to 0.25 ± 0.03 K.day⁻¹. The study underscores Antarctica as a key region for understanding aerosol impacts on climate and the importance of further research on aerosol sources and radiative effects. Sai Krishnaveni et al. (2023) applied Fuzzy C-Means clustering to classify aerosols over Gadanki, India, using 26 optical and microphysical parameters from Sky Radiometer data (2008–2020). Three distinct clusters were identified mixed-mode moderately absorbing, mixed-mode slightly absorbing, and coarse-mode slightly absorbing aerosols with robust classification accuracy of ~98.4%, retaining ~94.5% of records under 10% random perturbation. Seasonal patterns showed biomass burning aerosols dominating pre-monsoon, polluted marine aerosols during the monsoon, and polluted continental aerosols in winter and post-monsoon. The clusters corresponded closely to global aerosol types, confirming the effectiveness of FCM for reliable aerosol classification.

Filonchyk et al. (2024) analyzed sand and dust storms in northern China during March–April 2023, originating from the Gobi and Taklamakan deserts. Using satellite, ground-based, and reanalysis data, they found daily PM10 concentrations exceeding 1000 µg/m³ and AOD values above 1 across most northern regions. Aerosol properties indicated dominance of coarse-mode particles, with radiative forcing ranging from −48.5 to +2.7 W/m² at the top of the atmosphere and −180.8 to −66.6 W/m² at the surface, producing atmospheric heating rates of 1.8–3.7 K day⁻¹. The storms were mainly driven by cold fronts associated with low-pressure systems, lifting dust into the atmosphere and transporting it downwind. Chen et al. (2024) quantified the contributions of major Asian dust sources to spring 2023 dust events in China using the WRF-Chem model. Results showed that Mongolia was the dominant contributor, accounting for ~56% (82.7 μg m⁻³) of dust in North China and ~51% (15.9 μg m⁻³) in the Korean Peninsula. In southwest China, ~46% of dust originated from Inner Mongolia, while dust over the Tibetan Plateau mainly came from Xinjiang due to topographic blocking. The study highlights the role of surface soil conditions in driving frequent dust events and emphasizes the need for regional cooperation in dust mitigation. Jain et al. (2025) analyzed a pre-monsoon dust storm in May 2018 originating from the Thar Desert using satellite, reanalysis, and ground-based data. Dust column mass density increased 3–5 times during the event, reaching ~3.9 g m⁻² compared to background levels of ~0.2–0.6 g m⁻², with AOD > 2 indicating prolonged dust residence. HYSPLIT trajectories confirmed transport toward the Indo-Gangetic Plains, and strong associations with PM10/PM2.5 and meteorological variables highlighted severe air quality impacts.

**Comment 10.** Where is the MODIS uncertainty discussion?

**Response:** Thanks. A dedicated discussion on MODIS uncertainty has now been added in Section 5.3, highlighted in blue in lines 1055–1058, as shown below.

**5.3. Limitations**

Despite its strengths, the study faces several limitations. Real observational data were not available for all years, and daily records were limited, while data from neighboring countries were unavailable, restricting the assessment of transboundary dust contributions. In addition, accurate monthly wetland area data for the Shadegan Wetland were not consistently available, which constrained the evaluation of temporal variations in internal dust source.

Uncertainties associated with the data sources and analytical methods were considered to evaluate their potential influence on the study conclusions. AOD retrievals derived from the MODIS sensor are subject to known uncertainties, typically on the order of ±10–20%, arising from factors such as cloud contamination, surface reflectance over bright and arid regions, sensor viewing geometry, and calibration limitations. HYSPLIT trajectory simulations exhibit increasing positional uncertainty with time, reaching approximately ±50–100 km after 72 hours, which may affect the precise attribution of long-range dust transport pathways. Wetland area estimates based on the MNDWI index are prone to classification errors, particularly confusion between water, exposed soil, and sparse vegetation, leading to an estimated uncertainty of approximately ±5–10% in the calculated area. Statistical analyses, including correlation and clustering metrics, also inherently involve estimation errors.

**Comment 11.** Line 207: the symbol “—”what does it mean?

**Response:** Thanks. The “—” symbols were typographical errors and have been corrected throughout the manuscript.

**Reviewer #3**

**Comment 1.** In line no. 259-260: AOD definitions confuse standard concepts: AOD is misdefined as "measured in terms of the number of air particulate molecules per cubic kilometer". (AOD is dimensionless, representing optical extinction). Correct the sentence it.

Response: Thanks. The sentence has been corrected and is highlighted in green in lines 298–299, as shown below.

AOD is a dimensionless index that quantifies the extinction of solar radiation due to aerosols in the atmosphere.

**Comment 2.** Throughout the manuscript, PM2.5 and PM10 are mentioned at some places and PM2.5 PM10 at others. Please use these symbols uniformly in the whole manuscript.

**Response:** Thanks. The symbols PM2.5 and PM10 have been made consistent and corrected throughout the manuscript.

**Comment 3.** In line numbers 215, 251, 450, 483: correct the word dust ‘event’ to ‘events’.

**Response:** Thanks. The term “dust event” has been corrected to “dust events” in the mentioned lines.
