# Supplementary material for: Analysis and source identification of dust event in Khuzestan province using MODIS and SENTINEL satellite data and HYSPLIT model (2018–2022)
Source: PLoS One. 2026 May 7;21(5):e0348129. doi: 10.1371/journal.pone.0348129 (PMC13152170; doi:10.1371/journal.pone.0348129)
Supplement: Attachment — Submitted filename: Response to reviewers-editor.docx [file pone.0348129.s009.docx]

**Authors’ Response to the Reviewers’ Comments**

**Submission ID:** PONE-D-25-51186R2

**Title**: Analysis and Source Identification of Dust Event in Khuzestan Province Using MODIS and SENTINEL Satellite Data and HYSPLIT Model (2018–2022)

Dear Editor,

We sincerely thank you and the reviewers for your valuable time, constructive feedback, and positive evaluation of our manuscript. We are pleased that the revised version has been found to be technically sound and suitable for publication.

We have carefully considered the final editorial comment and have made the required minor revisions accordingly. The changes have been incorporated into the revised manuscript, and they are highlighted in the tracked version.

**Comment 1 (Editor):** Please include the appropriate attributions to Figure 2, 3 and Table 1. Please ensure that all tables, figures, and datasets are correctly attributed and their sources are appropriately cited.

**Response:** Thanks. We sincerely thank the editor for this valuable comment. The appropriate attributions have been added to Figure 2, Figure 3, and Table 1, and all relevant sources are now clearly indicated in the manuscript. The corresponding revisions have been highlighted in yellow for clarity.

For Figure 2, the source has been added in the caption as follows:

“Figure 2. Location and Land Cover Map of Khuzestan Province and Shadegan Wetland. Source: NASA Worldview (EOSDIS), MODIS True Color imagery (2024); processed and prepared by the authors.”

This modification can be found in lines 237–238. In addition, the attribution “(© NASA Worldview, EOSDIS, MODIS True Color imagery, 2024)” has also been included within the figure itself.

For Table 1, appropriate references have been added alongside the caption title as follows:

“TABLE 1. Air Quality Index Basics Pollution [38, 39],”

which can be found in line 307.

For Figure 3, the source has been added in the caption as follows:

“Figure 3. Regression analysis chart of AOD and PM₂.₅ and PM₁₀ for the years 2020–2022. Source: Derived from MODIS satellite data (NASA EOSDIS) and ground-based observations; processed and analyzed by the authors.”

This revision is reflected in lines 434–435, and the corresponding attribution has also been added within the figure.

Furthermore, all figures, tables, and datasets throughout the manuscript have been carefully reviewed to ensure that proper attribution and source citation are consistently applied.

**Figure 2.** Location and Land Cover Map of Khuzestan Province and Shadegan wetland. Source: NASA Worldview (EOSDIS), MODIS True Color imagery (2024); processed and prepared by the authors.

**TABLE 1.** Air quality index Basics Pollution [38, 39]

| **Levels of Concern** | **Values of Index AAI** | **Values of Index AOD** | **Values of Index AQI** |
| --- | --- | --- | --- |
| Air quality is satisfactory, and air pollution poses little or no risk. | <0 | <0.1 | 0-50 |
| Air quality is acceptable. However, there may be a risk for some people, particularly those who are unusually sensitive to air pollution. | 0-0.5 | 0.1-0.49 | 51-100 |
| Some members of the general public may experience health effects; members of sensitive groups may experience more serious health effects. | 0.5-2 | 0.5-1 | 101-200 |
| Health alert: The risk of health effects is increased for everyone. | 2-5 | >1 | 201-300 |
| Health warning of emergency conditions: everyone is more likely to be affected. | >5 | - | >300 |

**Figure 3.** Regression analysis chart of AOD and PM₂.₅ and PM_10_ for the year 2020-2022. Source: Derived from MODIS satellite data (NASA EOSDIS) and ground-based observations; processed and analyzed by the authors.

**Comment 2 (Editor):** Please take this opportunity to check that all tables, figures and datasets are correctly attributed and their sources cited appropriately.

**Response:** Thanks. All figures, tables, and datasets in the manuscript have been carefully reviewed to ensure proper attribution and accurate source citation. For figures derived from satellite data and the Google Earth Engine platform, appropriate source attributions have been added in accordance with the data usage and citation guidelines of the respective providers (e.g., NASA MODIS and related products). For other figures and tables based on ground-based observations, original datasets, or software-generated analyses, the existing attributions were deemed appropriate and have been retained. These revisions have been implemented throughout the manuscript and are highlighted in the revised version.

**Comment 3 (Editor):** Please carefully review the reference list to ensure that all citations are complete, accurate, and up to date. In particular, if any cited articles have been retracted, they should either be removed and replaced with relevant current references or clearly identified as retracted, with an appropriate explanation provided in the manuscript.

**Response:** Thanks. The entire reference list has been carefully reviewed to ensure that all citations are complete, accurate, and up to date, and that in-text citations are fully consistent with the reference list.

All references have been checked and properly formatted. One reference that was found to be unavailable (Hosseini Jani et al., 2020) has been removed and replaced with a relevant and valid source. The new reference has been added as Reference [14] in line 80 and is also reflected in the manuscript accordingly (line 1165):

Raji, H., Riahi, A., Borsi, S. H., Masoumi, K., Khanjani, N., AhmadiAngali, K., Goudarzi, G., & Dastoorpoor, M. (2020). Acute effects of air pollution on hospital admissions for asthma, COPD, and bronchiectasis in Ahvaz, Iran. International Journal of Chronic Obstructive Pulmonary Disease, 15, 501–514. https://doi.org/10.2147/COPD.S231317

In addition, Reference 25 has been corrected and properly formatted (line 1197):

Soleimani Sardo, F., & Krakauer, N. (2026). Dust modeling using MODIS data and WRF-Chem and HYSPLIT models (Case study of dust storm from December 16 to 20, 2016). Sustainable Earth Trends, 6(1), 55–65. https://doi.org/10.48308/set.2025.238019.1098

Furthermore, Reference 43 has been revised and updated accordingly (line 1251):

Yousefi Kebriya, A. R., Shiukhy Soqanloo, S., & Yousefi Kebriya, H. (2026). Monitoring and analysis of relationship between vegetation cover and air pollution using Sentinel and MODIS satellite data in Mazandaran Province. Iranica Journal of Energy & Environment, 17(2), 309–328. https://doi.org/10.5829/ijee.2026.17.02.08

All remaining references have also been thoroughly reviewed for completeness, accuracy, formatting consistency, and proper citation within the manuscript.

No retracted articles were identified among the cited references.

We appreciate the editor’s guidance in improving the quality and reliability of the manuscript.
